# Real-Time AI-Based Radiotherapy Planning for Nasopharyngeal Carcinoma: Development and Validation

**DOI:** 10.34133/cbsystems.0544

**Published:** 2026-05-18

**Authors:** Guangyu Wang, Xin Yang, Qianxi Ni, Junxiang Tang, Kailing Huang, Hailiang Guo, Xiaobo Jiang, Wenchao Diao, Hua Li, Yuxian Yang, Lecheng Jia, Yanfei Liu, Jiaxin Deng, Kang Zhang, Danyang Li, Xiaoyan Huang, Feng Jiang, Guanqun Zhou, Ying Sun

**Affiliations:** ^1^ State Key Laboratory of Oncology in South China, Guangdong Key Laboratory of Nasopharyngeal Carcinoma Diagnosis and Therapy, Guangdong Provincial Clinical Research Center for Cancer, Sun Yat-sen University Cancer Center, Guangzhou 510060, P. R. China.; ^2^ The Affiliated Cancer Hospital of Xiangya School of Medicine, Central South University/Hunan Cancer Hospital, Changsha 410013, P. R. China.; ^3^ Radiotherapy Business Unit, Shanghai United Imaging Healthcare Co. Ltd., Shanghai 201821, P. R. China.; ^4^ Jinan University Affiliated Guangdong Second Provincial General Hospital, Guangzhou 510317, P. R. China.; ^5^ Department of Oncology, The First Affiliated Hospital of Gannan Medical University, Jiangxi “Flagship” Oncology Department of Synergy for Chinese and Western Medicine, Jiangxi Provincial Unit for Clinical Key Oncology Specialty Development, Jiangxi Clinical Research Center for Cancer, Ganzhou 341000, P. R. China.; ^6^ Radiotherapy Laboratory, Shenzhen United Imaging Research Institute of Innovative Medical Equipment, Shenzhen 518048, P. R. China.; ^7^ Zhejiang Engineering Research Center for Innovation and Application of Intelligent Radiotherapy Technology, Wenzhou 325000, P. R. China.; ^8^ School of Biomedical Engineering, Southern Medical University, Guangzhou 510515, P. R. China.; ^9^ Department of Radiotherapy, Zhejiang Cancer Hospital, Hangzhou 310022, P. R. China.

## Abstract

**Background:** Online all-in-one (AIO) radiotherapy workflows enable same-day treatment by integrating simulation, planning, and delivery into a single session. However, for anatomically complex tumors such as nasopharyngeal carcinoma (NPC), generating high-quality plans within strict time constraints remains a major barrier to clinical adoption. **Methods:** We developed a deep-learning-based automated planning model specifically tailored for real-time NPC planning in the online AIO workflow. The model was trained on 890 patients and iteratively refined through 4 versions, incorporating innovations such as quantile loss, priority-based constraint encoding, and hybrid central processing unit–graphics processing unit acceleration. Model performance was benchmarked in a 5-center retrospective study, including 125 patients from the model development center and 120 patients from 4 external centers. It was then prospectively validated in 242 consecutively treated patients with NPC using a CT-linear accelerator-based AIO platform. **Results:** In the 5-center retrospective evaluation, artificial intelligence (AI)-generated plans achieved superior or comparable dosimetric quality relative to expert manual plans, despite variations in imaging, contouring, and prescription practices. In prospective deployment, 95% of plans were clinically accepted after a single optimization cycle, with a mean generation time of 3.5 min. All plans met target coverage criteria and passed both secondary dose verification and in vivo electronic portal imaging device analysis. **Conclusion:** This study represents the largest prospective validation to date of AI-based treatment planning for NPC, demonstrating real-time feasibility, robust generalizability, and consistent clinical quality. Our development-to-deployment framework supports the scalable adoption of AI-driven precision planning and provides a transferable model for intelligent radiotherapy across disease sites.

## Introduction

Nasopharyngeal carcinoma (NPC) is highly prevalent in East and Southeast Asia, with an incidence markedly higher than in Western countries. In China, the annual incidence reaches ~3.0 per 100,000, posing a major public health challenge due to its aggressive nature and anatomical complexity [1]. Most NPC cases are undifferentiated squamous cell carcinomas and are highly radiosensitive. Surgical resection is often limited by anatomy, so radiotherapy remains the primary treatment for early- and intermediate-stage disease [1–3]. Intensity-modulated radiation therapy and volumetric-modulated arc therapy have become standard modalities due to superior dose conformity and improved organs-at-risk (OAR) sparing [4–6]. However, traditional offline radiotherapy workflows often involve a 2- to 4-week delay between simulation and treatment initiation [7–9]. During this interval, tumor progression, weight loss, and anatomical changes can occur, making the initial planning computed tomography (CT) less representative at the time of delivery [10–12].

Online all-in-one (AIO) radiotherapy integrates simulation, contouring, planning and delivery into a single session, typically completed within 30 min. This condensed workflow is emerging as a practical alternative to traditional multistep pathways [13–15]. Real-time imaging and rapid plan generation enable same-day treatment based on up-to-date anatomy, minimizing geometric uncertainties and improving dose accuracy. The online AIO workflow markedly enhances treatment efficiency, precision, and patient experience. However, it imposes stringent requirements on plan quality, consistency, and computational speed. Meeting these requirements is particularly difficult in NPC, where sharp dose gradients and robust OAR sparing are essential.

Conventional planning is a manual, iterative process that requires repeated adjustments by experienced physicists. It may take hours or days depending on case complexity, and plan quality varies with individual expertise, effort invested, and institutional protocols [16–19]. Such variability can result in suboptimal dose distributions and inconsistent plan quality, undermining the reproducibility and effectiveness of radiotherapy. This inconsistency is fundamentally at odds with the speed and standardization required by the online AIO workflow. High-performance automated planning is therefore essential to rapidly generate consistent, high-quality plans with minimal manual input.

Automated planning has evolved from protocol-driven templates to data-driven artificial intelligence (AI) methods. Template-based approaches enforce standardization via fixed constraints but lack flexibility for patient-specific anatomical variations [20,21]. Knowledge-based planning improves adaptability using historical data and statistical models, enhancing planning efficiency and interplanner consistency [22,23]. However, knowledge-based planning still relies on curated datasets and manual tuning, which limit real-time responsiveness. In contrast, deep learning models can predict dose distributions or generate fluence maps directly from patient imaging and structure data, enabling plan generation within strict time limits [24,25]. Nonetheless, NPC presents unique challenges. Its anatomical complexity, proximity to critical structures, and stringent dose requirements can limit generalization from models trained on other disease sites [26–28]. Balancing target coverage and OAR sparing remains particularly challenging. Moreover, most deep-learning-based planning systems are still confined to research environments, with limited clinical integration due to engineering and workflow barriers [29,30].

Motivated by the anatomical complexity and real-time demands of online NPC radiotherapy, we developed an automated planning model tailored for true real-time clinical use. The model was trained on a large cohort and refined across 4 versions to meet clinical limits. We evaluated generalizability using an offline benchmark. This benchmark included a time-isolated cohort from the developing center and an external multicenter cohort from 4 independent centers. We then deployed the system prospectively in routine care. The primary outcomes were first-pass clinical acceptance, planning time, and target coverage. Secondary outcomes were OAR dose and delivery verification.

This work establishes a development-to-deployment framework for clinically integrated AI planning. It shows how technical innovation, when aligned with real-world clinical constraints, can enable precise and time-efficient real-time radiotherapy for complex cancers such as NPC.

## Materials and Methods

### Patient cohort

We developed and clinically validated an automated planning model for an online real-time AIO radiotherapy workflow in NPC. A total of 1,377 patient datasets were assembled. For model development, we used 890 cases from a single institution collected from 2020 January to 2021 June. These cases were managed by 4 senior attending physicians with 10 to 20 years of experience, who delineated the target volumes and organs at risk for their respective patients. Based on the planning CT and physician-approved structure sets, the clinical plans were manually generated by 6 medical physicists, with each case planned independently by a single physicist. One physicist had more than 3 years of experience, and the remaining physicists had 5 to 15 years of experience, including 2 associate senior physicists. Each plan was reviewed and approved by the responsible attending physician and then underwent a final plan review by 2 senior physicists with more than 20 and 25 years of experience. Disagreements were resolved by consultation with a third senior physicist. These clinically approved plans were used for training, validation, and testing of the model, including dose prediction and the CT-based Monte Carlo dose learning (CT-MCDL) module.

For offline multicenter evaluation, we used 245 cases, including 125 cases from the developing center and 120 cases from 4 independent external centers, with 30 cases contributed by each center. This 4-center dataset enabled an evaluation of the model’s generalizability. The offline evaluation cases were collected using the same data assembly and plan quality control procedures as the development cohort. In addition, a prospective cohort of 242 consecutive patients, treated from 2022 March to 2024 November, was enrolled to evaluate performance under real-time online conditions.

Across all datasets, eligible patients had nonmetastatic NPC and underwent definitive-intent radiotherapy (with or without systemic therapy) with a clinically approved treatment plan. For inclusion, we required the availability of planning CT, physician-approved target and OAR structures, the final approved plan, and the corresponding 3-dimensional (3D) dose distribution to enable standardized dosimetric evaluation. Patients were excluded if treatment was delivered with palliative or adjuvant intent, if there was prior head-and-neck radiotherapy (reirradiation), if metastatic disease was present at diagnosis, or if any of the required planning data were incomplete or not available for analysis. The same eligibility and data-completeness criteria were applied to the development cohort and the retrospective benchmark cohort, ensuring that comparisons were not confounded by missing structures, unavailable dose data, or nondefinitive treatment intent.

For the prospective cohort, eligibility additionally required that the patient’s initial radiotherapy plan be generated and delivered under the online AIO workflow, instead of the routine offline workflow. Consecutive patients scheduled for definitive radiotherapy or chemoradiotherapy in the online AIO workflow were enrolled after informed consent. Cases were excluded if study procedures were declined or if the online workflow could not be completed because of nonclinical interruptions. Baseline characteristics of the study cohorts are summarized in Table 1. Baseline distributions for the module-specific development splits, including training and held-out sets for dose prediction and CT-MCDL module, are provided in Table S1. The ethics committee of Sun Yat-sen University Cancer Center approved the study (XJS2022-060-01). Written informed consent was obtained for the prospective cohort.

**Table 1.** Baseline characteristics of the study cohorts

Characteristics	Model development cohort (*N* = 890)	Independent internal test cohort (*N* = 125)	Prospective validation cohort (*N* = 242)
**Sex**			
Male	561 (63.0%)	83 (66.4%)	153 (63.2%)
Female	329 (37.0%)	42 (33.6%)	89 (36.8%)
**Age**/years			
Median (range)	48 (18–81)	46 (19–82)	48 (23–81)
**KPS**			
70–80	0 (0)	0 (0)	0 (0)
90–100	890 (100%)	125 (100%)	242 (100%)
**Tumor category** ^a^			
T1	133 (14.9%)	9 (7.2%)	15 (6.2%)
T2	177 (19.9%)	16 (12.8%)	19 (7.9%)
T3	337 (37.9%)	83 (66.4%)	178 (73.6%)
T4	243 (27.3%)	17 (13.6%)	30 (12.4%)
**Nodal category** ^a^			
N0-1	563 (63.3%)	65 (52.0%)	155 (64.0%)
N2-3	327 (36.7%)	60 (48.0%)	87 (36.0%)
**Stage** ^a^			
I–II	233 (26.2%)	20 (16.0%)	22 (9.1%)
III–IV	657 (73.8%)	105 (84.0%)	220 (90.9%)
**Treatment modality**			
RT	113 (12.7%)	12 (9.6%)	29 (12.0%)
CCRT	322 (36.2%)	25 (20.0%)	83 (34.3%)
IC + CCRT	204 (22.9%)	70 (56.0%)	57 (23.6%)
CCRT + AC; IC + CCRT + AC	251 (28.2%)	18 (14.4%)	73 (30.2%)

KPS, Karnofsky performance score; RT, radiotherapy; CCRT, concurrent chemoradiotherapy; IC, induction chemotherapy; AC, adjuvant chemotherapy.

^a^ American Joint Committee on Cancer/Union for International Cancer Control, 8th edition

### Development of the final AI-based automated planning model

**Fig. 1.**
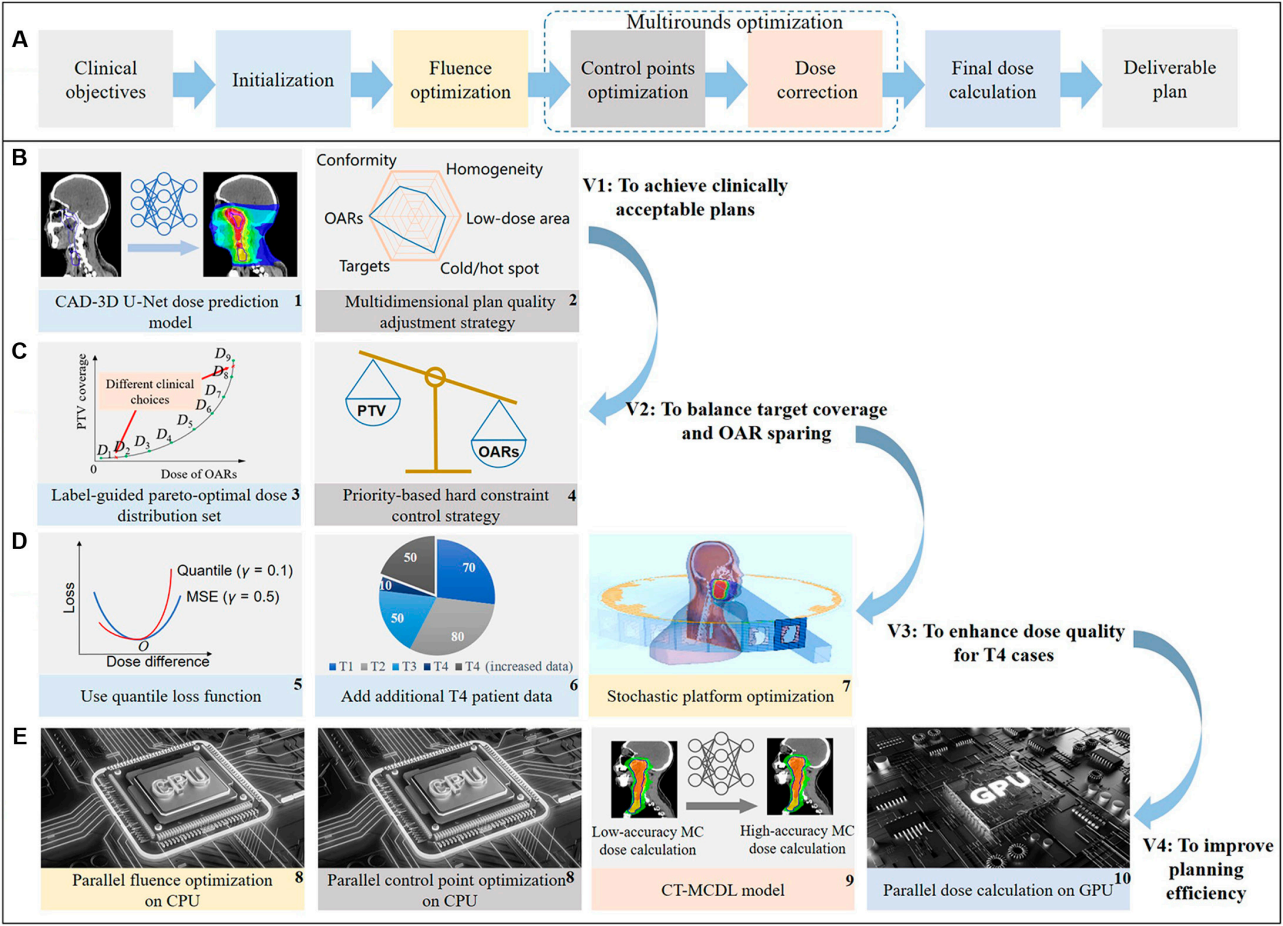
Iterative development and technical framework of the AI-based automated planning model. (A) Overview of the full pipeline for online AIO radiotherapy planning, from clinical prescription to deliverable plan. (B) Baseline model (V1): A CAD-3D U-Net-based dose prediction module combined with multidimensional plan adjustment. (C) V2 upgrades: introduction of label-guided pareto-optimal dose selection and a priority-based constraint mechanism to balance target coverage and OAR sparing. PTV, planning target volume. (D) V3 upgrades: improvements in robustness for complex T4 cases via quantile loss, dataset enrichment, and stochastic platform optimization. MSE, mean squared error. (E) V4 upgrades: computational acceleration through CPU-parallelized optimization, Monte Carlo dose learning, and GPU-based final dose calculation.

An AI-based automated planning model was developed specifically for this study to support a workflow that converts clinical dose objectives into deliverable treatment plans. Fig. 1A presents the overall automated planning framework. Fig. 1B shows the baseline algorithmic architecture (version 1 [V1]), which served as the foundation for later model iterations.

V1 consisted of 2 key components: (a) A dose prediction network based on a channel-attention densely connected 3D U-Net (CAD-3D U-Net) (Fig. 1B1) predicts the 3D dose distribution from planning CT images and structure contours [31]. The predicted dose is used as the initial reference for optimization, forming the core of the automated planning pipeline; (b) A multidimensional adjustment strategy (Fig. 1B2) dynamically adapts the dose distribution during optimization to better match clinical expectations. This dual-module architecture laid the groundwork for a functional and adaptable autoplanning model. Building on V1, the algorithm underwent 3 major iterations (V2 to V4; Fig. 1), each enhancing the model’s dosimetric quality or operational efficiency through specific technical strategies. V2 (Fig. 1C) improved planning quality by introducing a label-guided prioritization mechanism (Fig. 1C3), which incorporated user-defined clinical priorities directly into the optimization process, and a priority-based hard constraint control mechanism (Fig. 1C4), which adaptively adjusted the objective weights of planning target volumes and OARs according to the assigned priorities. This allowed the model to generate a set of pareto-optimal dose distributions, reflecting different trade-offs between tumor coverage and OAR sparing. The model then selected the optimal distribution based on predefined clinical thresholds and trade-off preferences.

V3 (Fig. 1D) focused on enhancing model robustness for advanced T4 stage NPC cases through 3 upgrades: (a) incorporation of a quantile loss function (Fig. 1D5) to reduce prediction sensitivity to outliers [32,33]; (b) expansion of the training dataset with 50 additional T4 cases (Fig. 1D6) to improve generalizability; and (c) replacement of the conventional fluence map optimization algorithm with a stochastic platform optimization method to improve dose conformity and homogeneity (Fig. 1D7) [34]. The final V4 (Fig. 1E) aimed to meet the demands of real-time application in online workflows by introducing 3 key computational enhancements: (a) a CT-MCDL module (Fig. 1E9), enabling accurate dose estimation with minimal time cost [35]; (b) parallelized central processing unit (CPU)-based optimization during the iterative objective function calculation phase (Fig. 1E8); (c) graphics processing unit (GPU)-accelerated dose computation in the final dose calculation phase (Fig. 1E10). These improvements substantially reduced planning time and enhanced the model’s clinical feasibility for integration into time-sensitive workflows.

Through the integration of several targeted strategies, these iterations improved both the plan quality and the computational efficiency of the automated planning model in a consistent manner. Details of the implementation environment and training setup for the deep learning components, including software frameworks, hardware configurations, training parameters and dataset composition, are provided in Section S1.

### Multicenter retrospective validation against manual plans

To assess cross-institutional performance of the automated planning model, we conducted an offline multicenter evaluation using 245 NPC cases. The cohort included 125 cases from the developing center and 120 cases from 4 independent external centers, with 30 cases from each external center. Cases from each center spanned T1 to T4 disease. All cases were assembled using consistent inclusion criteria and the same plan quality control framework as the development cohort to ensure comparable inputs for dosimetric evaluation.

Manual plans were generated by experienced medical physicists following routine clinical practice at each institution and served as the reference for evaluating whether the automated plans could reach expert-level quality across heterogeneous clinical settings. For each case, manual and AI plans were generated from the same imaging, structure sets, and prescription and were computed under the same planning configuration within that case comparison to avoid confounding from technique selection or dose calculation settings. Manual plans were iteratively refined to meet local clinical goals. To better reflect routine practice and reduce reliance on any single planning style, manual plans at each center were generated by multiple physicists, and each case was completed independently by a single physicist. AI-generated plans were created fully automatically without manual intervention. Plan quality and consistency were then compared across centers to evaluate portability of the automated planning model under differences in contouring habits, planning preferences, and dose protocols. To further characterize intrinsic manual planning variability and to contextualize AI–manual differences, we also performed an interplanner analysis on a representative case.

### Clinical implementation of the online AIO radiotherapy workflow

**Fig. 2:**
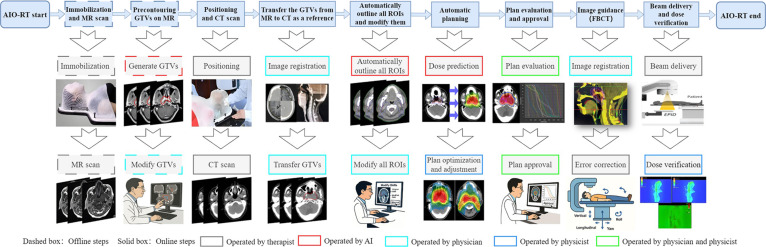
Implementation workflow of real-time online AIO radiotherapy (RT) for NPC. Workflow steps include patient immobilization, image acquisition via integrated CT-linac (FBCT), AI-assisted segmentation of targets and OARs, automated planning, physician review, QA verification, and same-session beam delivery. GTVs, gross tumor volumes.

We deployed the online AIO workflow on an integrated CT-linear accelerator (CT-linac) platform (uRT-linac 506c, United Imaging Healthcare Co. Ltd., Shanghai, China). The system acquires kilovoltage fan-beam CT (FBCT) in the treatment room and supports planning and delivery in one session [13,36]. Before each online session, patients undergo immobilization and magnetic resonance imaging (MRI) acquisition (Fig. 2). At treatment, FBCT images are obtained and registered with MRI to guide automated delineation of all target volumes and OARs. After physician-led review of regions of interest (ROIs), auxiliary structures are generated using scripting tools (see Section S2 and Figs. S1 to S6). The AI-based automated planning model then generates a treatment plan, which is reviewed and, if necessary, adjusted by the physician and physicist. Beam delivery proceeds following image-guided position verification.

Dosimetric quality assurance (QA) includes independent dose recalculation via uAssureTx (United Imaging Healthcare Co. Ltd., Shanghai, China) and in vivo verification using electronic portal imaging devices (EPIDs) and the integrated QA system of the treatment platform (see the “Dosimetric QA” section for details).

### Prospective clinical evaluation in the online setting

We prospectively assessed feasibility, plan quality, and dosimetric safety of the final model within the online AIO workflow, encompassing the full pipeline from prescription to optimization, evaluation, and QA.

#### Dose prescription and planning objectives

The dose objectives and optimization parameters used in this study were determined on the basis of institutional experience to ensure clinically acceptable plan quality. For each target volumes (e.g., PGTVp [planning target volume of gross tumor at the primary site and the involved retropharyngeal lymph nodes], PGTVn [planning target volume of involved cervical lymph nodes], PCTV1 [planning target volume of high-risk clinical target volume], and PCTV2 [planning target volume of low-risk clinical target volume]) and OARs, specific dose coverage goals and constraint limits were defined to guide automated plan generation.

All constraints were implemented using a unified optimization strategy. Variations in clinical priority were represented by different priority levels and internal constraint weights. Higher-priority structures, such as the brainstem and spinal cord, were assigned stronger weights to emphasize their protection during optimization, while lower-priority ROIs were treated with greater flexibility to allow reasonable trade-offs. Planning parameters such as dose calculation algorithm, dose grid resolution, and number of iterations were standardized across all cases. A comprehensive summary of dose objectives, constraint types, assigned priorities, and planning settings is provided in Table 2.

**Table 2.** Dose prescriptions, optimization priorities, and planning constraints for targets and OARs

Protocol	Items			
Prescription	PGTVp, 6,996 cGy/33 Fr; PGTVn_L/R, 6,600 or 6,996 cGy/33 Fr; PCTV1, 6,006 cGy/33 Fr; PCTV2, 5,412 cGy/33 Fr			
Optimization objective	**ROIs**	**Goal**	**Value/cGy**	**Priority**
	**Targets**			
	PGTVp	Prescription	6,996	2
		*D*_max_ ^a^	≤7,400	2
	PGTVn_L/R	Prescription	6,600 or 6,996	2
		*D*_min_ ^b^	≥6,650 or 7,050	1
		*D*_max_	≤7,000 or 7,400	2
	PCTV1	Prescription	6,006	2
		*D*_min_	≥6,050	1
		*D*_max_	≤6,700	2
	PCTV2	Prescription	5,412	2
		*D*_min_	≥5,450	1
		*D*_max_	≤5,800	2
	**OARs**			
	BrainStem	*D*_max_	≤7,000	1
		*V*_60Gy_ ^c^	≤5%	1
	BrainStem 3 mm	*V*_60Gy_	≤10%	1
	SpinalCord	*D*_max_	≤4,500	1
	TemporalLobe_L/R	*V*_70Gy_ ^d^	≤2%	1
		*V*_60Gy_	≤5%	1
	OpticNerve_L/R	*D*_max_	≤7,000	2
	Lens_L/R	*D*_0.03cc_ ^e^	≤600	2
	Chaism	*D*_0.03cc_	≤5,400	2
	Pituitary	*D*_mean_ ^f^	≤5,400	2
	Eye_L/R	*D*_0.03cc_	≤5,400	2
		*D*_mean_	≤3,500	2
	Parotid_L/R	*D*_mean_	≤2,600	3
		*V*_30Gy_ ^g^	≤50%	3
	Submandibular_L/R	*D*_mean_	≤3,500	3
	OralCavity	*D*_mean_	≤4,000	3
	Mandible_L/R	*D*_2%_ ^h^	≤7,000	3
	Thyroid	*D*_mean_	≤3,000	3
	**Auxiliary structures**			
	Z40	*D*_max_	≤3,900	1
	Z54_total	*D*_max_	≤5,350	2
	Z54_lobe	*D*_max_	≤5,400	2
	6996-Brainstem	*D*_min_	≥7,050	1
	Parotid_Norm_L/R	*D*_mean_	≤5,000	1
	Ring	*V*_60Gy_	<=15%	2
Optimization settings	VMAT; two full arcs			
	Monte Carlo algorithm; 30 iterations; dose grid: 3 mm			
	Plan normalization: 100% prescription dose covers 98.5% of PGTVp			

Note: (a) Constraints for target volumes not included in the actual prescription are omitted from optimization. (b) Auxiliary structure generation is detailed in the Supplementary Section 2. (c) The “Prescription” objective is intended to ensure that each constrained target volume meets the predefined dose coverage criteria. Specifically, the required coverage is 97.5% for PGTVp and 98.5% for the remaining targets. (d) Priority is assigned using 3 levels. Level 1 indicates the highest priority, with a weight greater than that of the targets. Level 2 represents a medium priority, with a weight equal to that of the targets. Level 3 denotes the lowest priority, with a weight lower than that of the targets.

^a^ Maximum dose to the volume.

^b^ Minimum dose to the volume.

^c^ Percent of volume receiving ≥60 Gy.

^d^ Percent of volume receiving ≥70 Gy.

^e^ Dose received by 0.03 cm^3^ of the volume.

^f^ Mean dose to the volume.

^g^ Percent of volume receiving ≥30 Gy.

^h^ Dose received by 2% of the volume.

#### Plan evaluation criteria

To ensure a standardized and clinically meaningful evaluation of plan quality, both target coverage and OAR sparing were assessed using guideline-recommended dosimetric parameters [37]. For target volumes, the primary evaluation metrics included the volume percentages receiving at least 100%, 98%, and 95% of the prescribed dose (*V*_100%_, *V*_98%_, and *V*_95%_), as well as the dose received by 100% and 98% of the target volume (*D*_100%_ and *D*_98%_). These parameters reflect dose coverage within the target.

For OARs, evaluation focused on the maximum dose received by the most irradiated 0.03 cm^3^ (*D*_0.03cc_), which is particularly relevant for serial structures such as the brainstem and spinal cord. In addition, the mean dose (*D*_mean_), maximum point dose (*D*_max_), and near-maximum dose to 2% of the volume (*D*_2%_) were assessed to evaluate overall dose burden and potential toxicity. These dosimetric parameters were used consistently across all plans to assess and compare the quality and safety of the automatically and manually generated treatment plans.

#### Dosimetric QA

To ensure the accuracy and safety of dose delivery, this study implemented 2 levels of dose verification through pretreatment independent dose calculation and in vivo dose monitoring during treatment. Pretreatment verification was performed using an independent dose calculation software (uAssureTx, United Imaging Healthcare Co. Ltd., Shanghai, China). Upon plan approval, treatment plans were automatically exported from the treatment planning system to the uAssureTx software, where a secondary dose computation was conducted using a separately commissioned algorithm. Gamma analysis was conducted using 3 criteria: A dose difference of 3% and distance to agreement of 3 mm (3%/3 mm), 3%/2 mm, and 2%/2 mm, with a 10% global dose threshold. A gamma passing rate greater than 95% under the 3%/3-mm criterion was required for plan acceptance.

In vivo dosimetry was conducted using an integrated amorphous silicon EPID (XRD1642, Varex Imaging Corporation, UT, USA). Exit fluence was captured during each treatment fraction and analyzed using the built-in QA module of the treatment delivery system. Delivered dose was reconstructed using Monte Carlo algorithms and daily FBCT images acquired immediately before treatment to account for the patient’s actual anatomy. 3D gamma analysis was performed under the same criteria as pretreatment verification. An action level of 90% gamma passing rate under the 3%/3-mm criterion was used for in vivo verification, recognizing that in vivo measurements are more sensitive to multiple sources of error, including anatomical variations, interfraction setup deviations, intrafraction motion, and machine-related uncertainties such as multileaf collimator positioning and output calibration [15,38,39]. This 2-tiered verification strategy provided robust QA for both the accuracy of plan generation and the fidelity of dose delivery within the online AIO workflow.

### Statistical analysis

Statistical comparisons between AI-generated and manual plans were conducted using the Wilcoxon signed-rank test for paired data. A 2-tailed *P* < 0.05 was considered statistically significant. Analyses were performed in SPSS (version 27.0, IBM, Armonk, NY).

## Results

### Gains with iterative model development

**Fig. 3.**
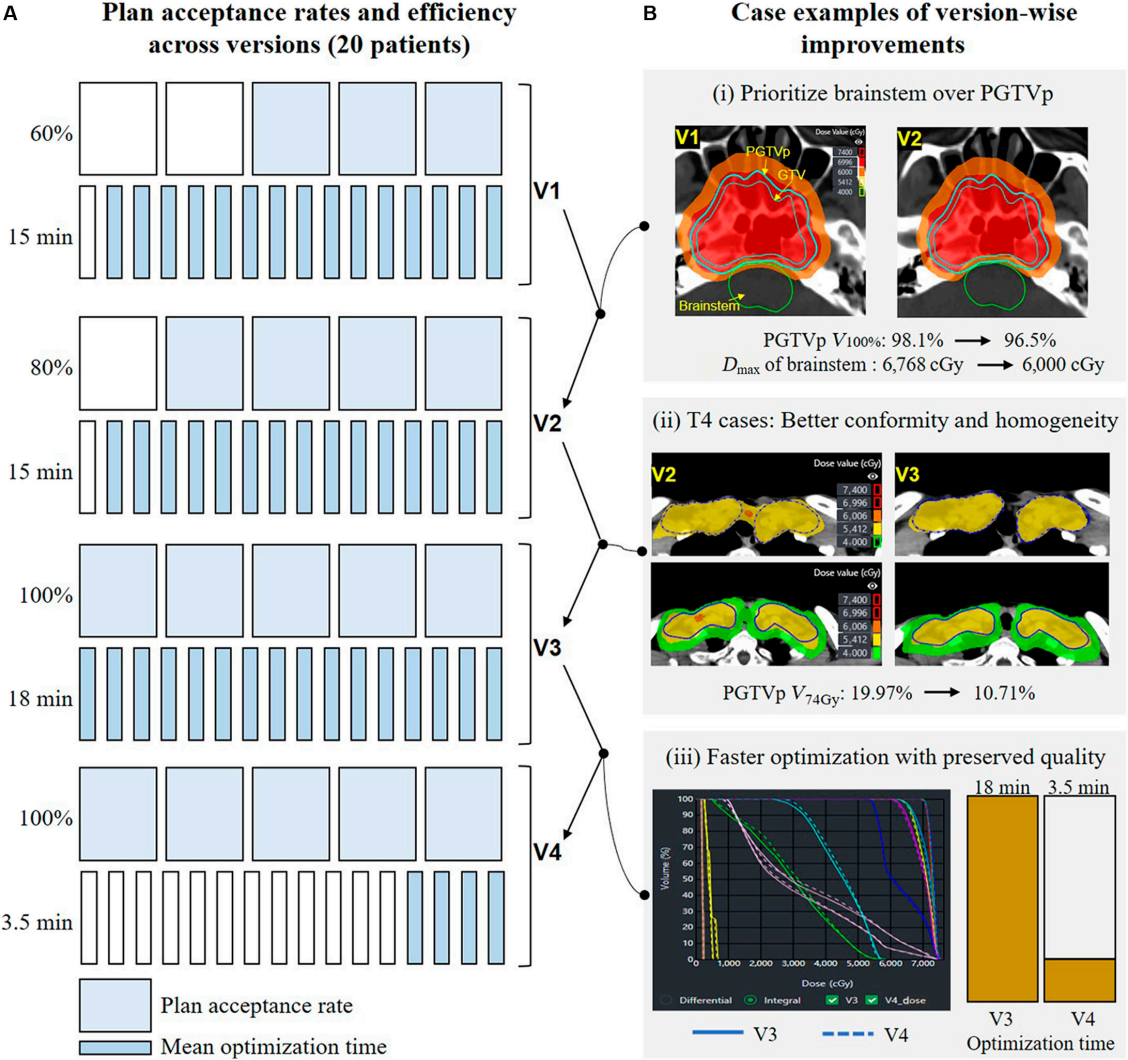
Performance evolution across 4 model versions (V1 to V4). (A) Plan acceptance rates and average optimization time across 20 NPC cases, assessed by a senior radiation oncologist. (B) (i) V1 versus V2: improved brainstem sparing without compromising PGTVp coverage. (ii) V2 versus V3: enhanced dose conformity and homogeneity in a T4 lesion. (iii) V3 versus V4: drastic reduction in optimization time while maintaining dose quality.

We compared V1 to V4 in 20 patients across tumor stages (T1 to T4). Plan acceptance rose from 60% in V1 to 80% in V2 and reached 100% in V3 and V4 (Fig. 3A). Acceptance meant that all institutional constraints were met without manual edits and were confirmed by a senior radiation oncologist. In parallel, the average plan optimization time decreased from 15 to 18 min in V1 to V3 to 3.5 min in V4, reflecting substantial efficiency gains in the final version.

Visual and quantitative comparisons across representative cases further highlight key improvements in model performance. In case 1 (Fig. 3Bi), V1 achieved adequate PGTVp coverage (*V*_100%_ > 95%) but failed to limit brainstem dose, with *D*_max_ exceeding 6,768 cGy. In contrast, V2 effectively reduced brainstem *D*_max_ to 6,000 cGy while preserving target coverage. In case 2 (Fig. 3Bii), representing a T4 lesion, V3 yielded multiple improvements compared to V2, including better dose conformity, enhanced dose homogeneity, improved hotspot control (*V*_74Gy_ reduced from 19.97% to 10.71%), and more effective midline dose modulation within the PCTV2 region. Case 3 (Fig. 3Biii) demonstrated the acceleration advantage of V4, reducing the average optimization time from 18 to 3.5 min while maintaining comparable dosimetric quality.

These results highlight the stepwise maturation of the model from dosimetric robustness to workflow efficiency, culminating in a clinically deployable version capable of fast and reliable plan generation.

### Multicenter dosimetric validation

#### Time-isolated internal benchmark at the developing center

**Fig. 4.**
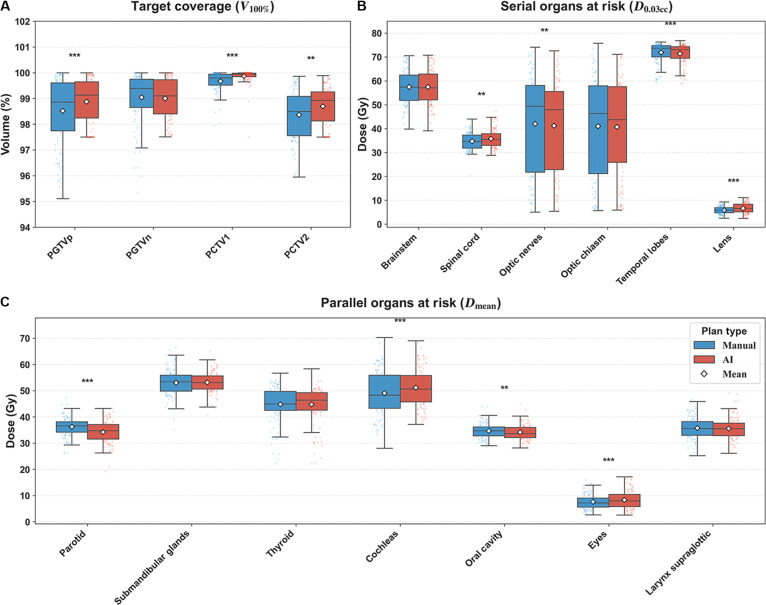
Pairwise dosimetric comparison between AI-generated and manual plans in the independent internal test cohort. (A) Target coverage assessed by the percentage of volume receiving 100% of the prescription dose (*V*_100%_). (B) Near-maximum dose (*D*_0.03cc_) for serial OARs. (C) *D*_mean_ for parallel OARs. The box plots illustrate the distribution of dosimetric metrics. The horizontal black line denotes the median, the white diamond indicates the mean, and the box boundaries represent the interquartile range. Statistical significance is defined as **P < 0.01 and ***P < 0.001.

The performance of the automated planning model was assessed through a time-isolated internal benchmark (*n* = 125). Fig. 4 visually compares the dosimetric metrics of AI-generated and manual plans, while Table S2 reports the dosimetric values for both planning methods. Both the AI and manual plans met the coverage requirements for the targets, with the AI plan showing significant advantages in the coverage of PGTVp, PCTV1, and PCTV2. In addition, the AI model demonstrated superior robustness. As shown in Fig. 4A, the distribution of AI plans exhibited significantly narrower interquartile ranges across all targets, indicating reduced plan variability and higher standardization.

In terms of OAR sparing, the AI plans maintained clinically acceptable doses for all key organs, although organ-specific trade-offs were observed. For serial OARs, the AI plan showed lower *D*_0.03cc_ values for the optic nerves and temporal lobes, while the spinal cord and lenses exhibited slightly higher doses. For parallel OARs, the mean dose varied by structure. The AI plan reduced the *D*_mean_ for the parotid glands and oral cavity while increasing the *D*_mean_ for the eyes and cochleae. However, these differences were generally small and remained within limits. Overall, the internal benchmark demonstrated that in a time-isolated setting, the automated workflow provided stable plan quality, high target coverage, and OAR protection comparable to manual planning.

#### External multicenter benchmark across 4 independent centers

**Fig. 5.**
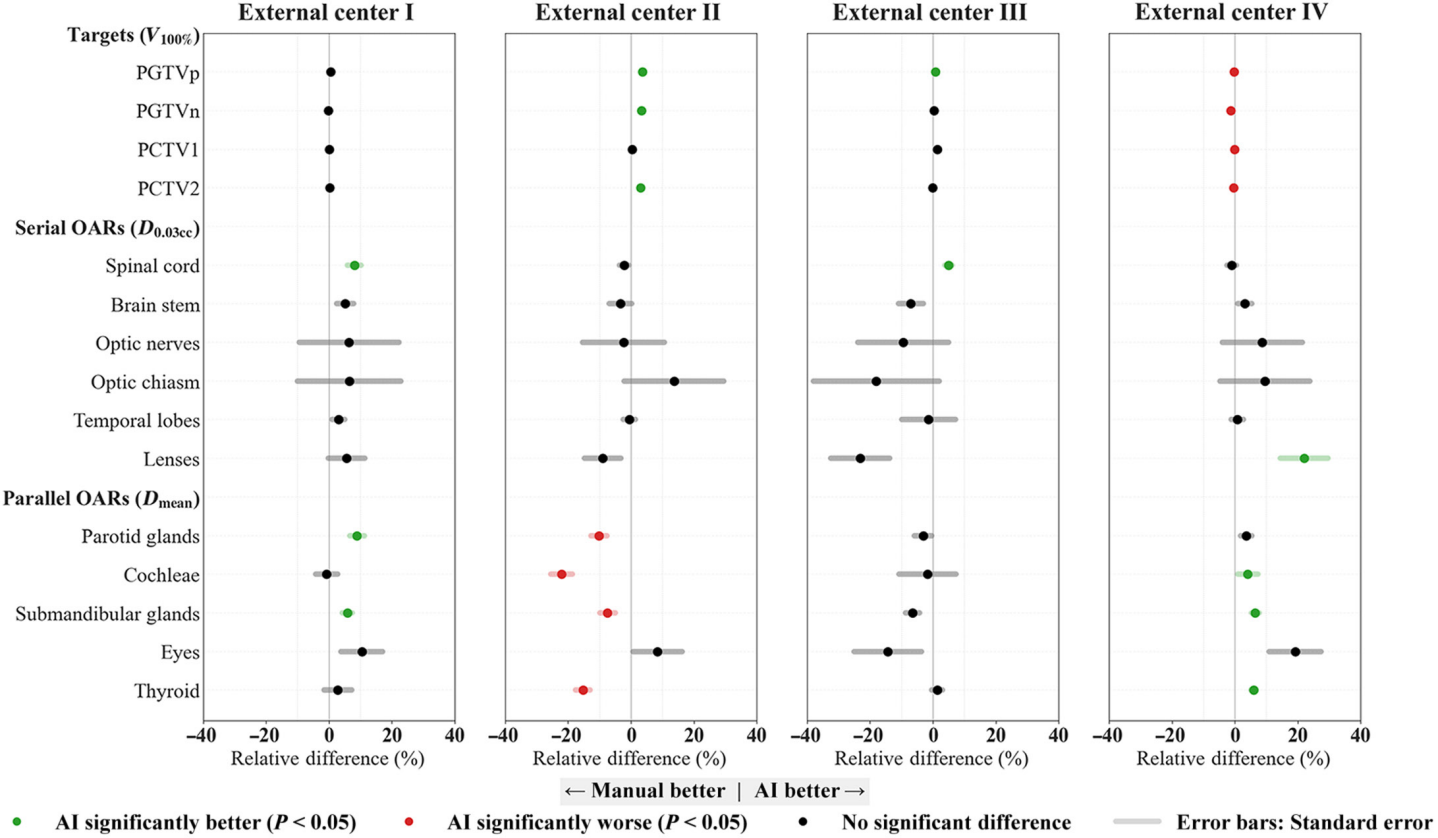
Quantitative dosimetric comparison between AI-generated and manual plans across 4 external validation centers. Panels summarize target coverage (*V*_100%_) and OAR dose metrics (serial organs *D*_0.03cc_ and parallel organs *D*_mean_). For each end point, the horizontal axis shows the paired relative difference, expressed as percent change, with positive values indicating better AI performance. For targets, percent change was computed as (AI − manual)/manual. For OARs, percent change was computed as (manual − AI)/manual, so that positive values consistently favor AI. The dots represent the mean relative difference, and the shaded horizontal bars represent the standard error. Green dots denote metrics where AI plans were statistically significantly better than manual plans (*P* < 0.05), while red dots denote significantly worse performance (*P* < 0.05). Black dots indicate no statistically significant difference.

A total of 120 patients were enrolled from 4 independent centers, with 30 cases per center, to evaluate the generalizability and robustness of the proposed automated planning model. Fig. 5 presents the relative dosimetric differences between AI-generated and manual plans, and Table S3 reports the dosimetric metrics for both approaches. The AI model showed reliable performance in maintaining target coverage, with results that were noninferior or superior to manual planning. This was most evident at center II, where AI plans achieved a statistically significant improvement in coverage. At center IV, target coverage in AI plans was slightly lower than that in manual plans, but the absolute difference was only about 1%. Given that most OAR metrics favored AI at this center, AI planning still demonstrated overall better performance. These findings indicate that the AI model can maintain high target coverage across diverse clinical settings.

For OAR sparing, AI plans showed an overall advantage. At centers I and IV, AI reduced doses for most OARs, including statistically significant reductions for the parotid glands, spinal cord, and submandibular glands at center I, and for the cochleae, submandibular glands, lenses, and thyroid at center IV. Center- and organ-specific differences were also observed. At center II, a clear trade-off pattern emerged. The AI model prioritized target coverage, which was accompanied by significantly higher doses to the parotid glands, cochleae, submandibular glands, and thyroid compared with manual plans. In contrast, the AI model emphasized OAR sparing at center IV while accepting a small compromise in target coverage. Centers I and III showed a more balanced performance profile. These differences likely reflect the model’s global optimization strategy. When geometric trade-offs are unavoidable, the model tends to prioritize target coverage and the protection of high-risk OARs rather than lower-priority structures. It may also relax dose constraints for some OARs when their limits are already met, thereby freeing optimization capacity for structures that still violate constraints. Institutional planning preferences may further contribute to these patterns. For example, manual plans at center II consistently prioritized OAR sparing even at the expense of target coverage (Table S3). In addition, manual planning often involves more iterative adjustments, enabling further fine-tuning of individual OAR constraints, which may explain localized improvements in specific end points.

Overall, the model remained applicable across different imaging protocols, contouring practices, and prescription dose schemes. It delivered stable plan quality, with strong target coverage and competitive OAR sparing. Section S4 further provides a representative T3N1M0 case comparing 3 independent manual plans with the AI plan. The results show that AI plan metrics remained within the range of the manual plans, as reported in Table S4 and Fig. S7.

### Prospective clinical performance in the online workflow

#### Feasibility and efficiency

We enrolled 242 patients. A total of 237 patients (97.9%) completed online planning and treatment. Five were excluded because of workflow interruptions: segmentation delays (*n* = 2), treatment planning system crashes (*n* = 2), and a network failure (*n* = 1).

Among the 237 evaluable cases, 225 plans (94.9%) were accepted after a single automated run. Twelve plans (5.1%) required reoptimization due to target overdose (*n* = 7), OAR violations (*n* = 3), or minor contour edits (*n* = 2).

Total planning time, including segmentation, priority setup, optimization, and dose calculation, averaged 6.5 min (range, 3.2 to 17.5). The median was 5.85 min. These results show the system is feasible for real-time use in routine care.

#### Dosimetric performance

A comprehensive dosimetric evaluation was performed for the 237 patients in the prospective cohort who successfully completed the online AIO workflow. Table 3 reports the quantitative results for the overall population and includes analyses stratified by tumor stage.

**Table 3.** Dosimetric evaluation in the prospective cohort overall and stratified by tumor stage

ROIs	Metrics	Criteria /cGy	Dosimetric results/cGy			
			All cases (*n* = 237)	T1–T2 cases (*n* = 34)	T3 cases (*n* = 178)	T4 cases (*n* = 25)
**Targets**						
PGTVp	*V*_100%_ ^a^	≥95%	99.4% ± 0.7%	99.7 ± 0.4	99.4 ± 0.6	98.9 ± 1.1
	*D*_100%_ ^b^	/	6,787.9 ± 189.8	6,873.8 ± 117.8	6,784.5 ± 176.5	6,702.1 ± 293.6
	*D*_98%_ ^c^	/	7,077.8 ± 52.8	7,097.9 ± 49	7,079.1 ± 48	7,042.3 ± 72.9
PGTVn	*V*_100%_	≥95%	99.3% ± 1.0%	99.6 ± 0.6	99.3 ± 1.1	99 ± 1
	*V*_98%_ ^d^	/	99.9% ± 0.2%	100 ± 0.1	99.9 ± 0.3	99.9 ± 0.2
	*V*_95%_ ^e^	/	100% ± 0%	100 ± 0	100 ± 0	100 ± 0
PCTV1	*V*_100%_	≥95%	99.9% ± 0.1%	100 ± 0.1	99.9 ± 0.1	99.9 ± 0.1
	*D*_100%_	/	5,895.4 ± 116.1	5,922.4 ± 69.6	5,896.7 ± 114.2	5,851.7 ± 161.1
	*D*_98%_	/	6,235.9 ± 52.0	6,227 ± 60.2	6,235.5 ± 50.2	6,250.7 ± 52.9
PCTV2	*V*_100%_	≥95%	97.7% ± 0.3%	97.6 ± 0.2	97.7 ± 0.3	97.8 ± 0.4
	*D*_100%_	/	4,624.1 ± 192.6	4,665.8 ± 157.6	4,619.9 ± 194.4	4,601.1 ± 218.8
	*D*_98%_	/	5,394.9 ± 22.5	5,388.9 ± 10.6	5,394.7 ± 22.1	5,404 ± 32.6
**Organs at risk**						
Spinal cord	*D*_0.03cc_ ^f^	≤5,000	3,181.3 ± 212.4	3,179.7 ± 219.5	3,185.1 ± 204.6	3,155.5 ± 261.9
Brain stem	*D*_0.03cc_	≤6,000	5,386.1 ± 516.4	5,102.7 ± 445.3	5,406.2 ± 475.9	5,604.1 ± 719.7
Temporal lobes	*D*_0.03cc_	≤7,200	6,922.3 ± 440.1	6,667.2 ± 424.7	6,949.5 ± 427.4	7,052.7 ± 445.9
Optic chiasm	*D*_0.03cc_	≤6,000	2,832.2 ± 1,610.3	2,392.7 ± 1,481.2	2,750.5 ± 1,530.5	3,983.1 ± 1,872.7
Optic nerves	*D*_0.03cc_	≤6,000	2,762.2 ± 1,607.8	2,513 ± 1,433.3	2,632.5 ± 1,579.3	4,015.3 ± 1,522.5
Eyes	*D*_mean_ ^g^	<3,500	585.7 ± 170.2	568.6 ± 136.2	573.5 ± 172.7	695.3 ± 156.7
Lenses	*D*_0.03cc_	≤600	501.9 ± 139.9	485.8 ± 102.6	497.3 ± 148.1	555.6 ± 108.5
Cochleae	*D*_mean_	<5,500	4,821.6 ± 734.9	4,456.6 ± 694.5	4,850 ± 686.3	5,084 ± 959
Parotid glands	*D*_mean_	<3,000	3,135.7 ± 476.9	3,043.6 ± 372.4	3,144.1 ± 467.1	3,192.9 ± 643.6
Submandibular glands	*D*_mean_	<3,500	5,150.2 ± 339.0	5,187.5 ± 359.3	5,161.7 ± 332.4	5,020.4 ± 345.1
Pituitary	*D*_max_ ^h^	<6,000	5,508.9 ± 1,167.4	5,429.6 ± 1,100.4	5,457.9 ± 1,160.8	5,978.1 ± 1,236.8
Thyroid	*D*_mean_	<4,500	4,499.2 ± 452.3	4,371.3 ± 648.9	4,547.2 ± 364.2	4,317 ± 636
Mandible	*D*_0.03cc_	<6,500	6,407 ± 588.5	6,406.9 ± 738.5	6,434.5 ± 560.7	6,209.5 ± 557
Temporomandibular joints	*D*_2%_ ^i^	<7,500	4,952.3 ± 786.8	4,544.8 ± 651.7	4,990.8 ± 772.7	5,196.9 ± 889.2

All data are presented as means ± SD.

^a^ Percent of volume receiving ≥100% of the prescribed dose.

^b^ Dose received by100% of the target volume.

^c^ Dose received by 98% of the target volume.

^d^ Percent of volume receiving ≥98% of the prescribed dose.

^e^ Percent of volume receiving ≥95% of the prescribed dose.

^f^ Dose received by 0.03 cm^3^ of the volume.

^g^ Mean dose to the volume.

^h^ Maximum dose to the volume.

^i^ Dose received by 2% of the volume.

Overall, AI plans achieved excellent and consistent target coverage. The mean *V*_100%_ values were 99.4% for PGTVp, 99.3% for PGTVn, 99.9% for PCTV1, and 97.7% for PCTV2. All other target coverage end points also met the institutional thresholds (>95%), indicating close adherence to planning objectives. Tumor stage stratification showed consistent performance. The mean PGTVp coverage was 99.7% in the T1 to T2 subgroup. Coverage decreased slightly to 98.9% in the T4 subgroup, with greater variability, but remained well above the 95% clinical acceptability threshold, which is consistent with the expected increase in anatomic complexity. The remaining target volumes showed similarly stable coverage across all strata, supporting reliable first-pass plan quality across disease stages.

OAR sparing met clinical expectations for most critical structures. The mean *D*_0.03cc_ values were 5,386.1 cGy for the brain stem, 3,181.3 cGy for the spinal cord, 2,762.2 cGy for the optic nerves, and 501.9 cGy for the lenses. Mild dose exceedances were observed only for the submandibular glands (mean, 5,150.2 cGy) and parotid glands (mean, 3,135.7 cGy), which is consistent with their proximity to high-dose target volumes. Stratified results were generally consistent with the overall findings. As tumor volume increased, OAR doses rose with advancing tumor stage and showed greater dispersion. For example, the optic chiasm dose increased from 2,392.7 cGy ± 1,481.2 cGy in the T1 to T2 subgroup to 3,983.1 cGy ± 1,872.7 cGy in the T4 subgroup. Despite these increases, doses in advanced cases remained within safety standards. Together, these results indicate that the automated planning model can balance tumor control and normal tissue toxicity across different disease stages.

#### Dose verification results

**Fig. 6.**
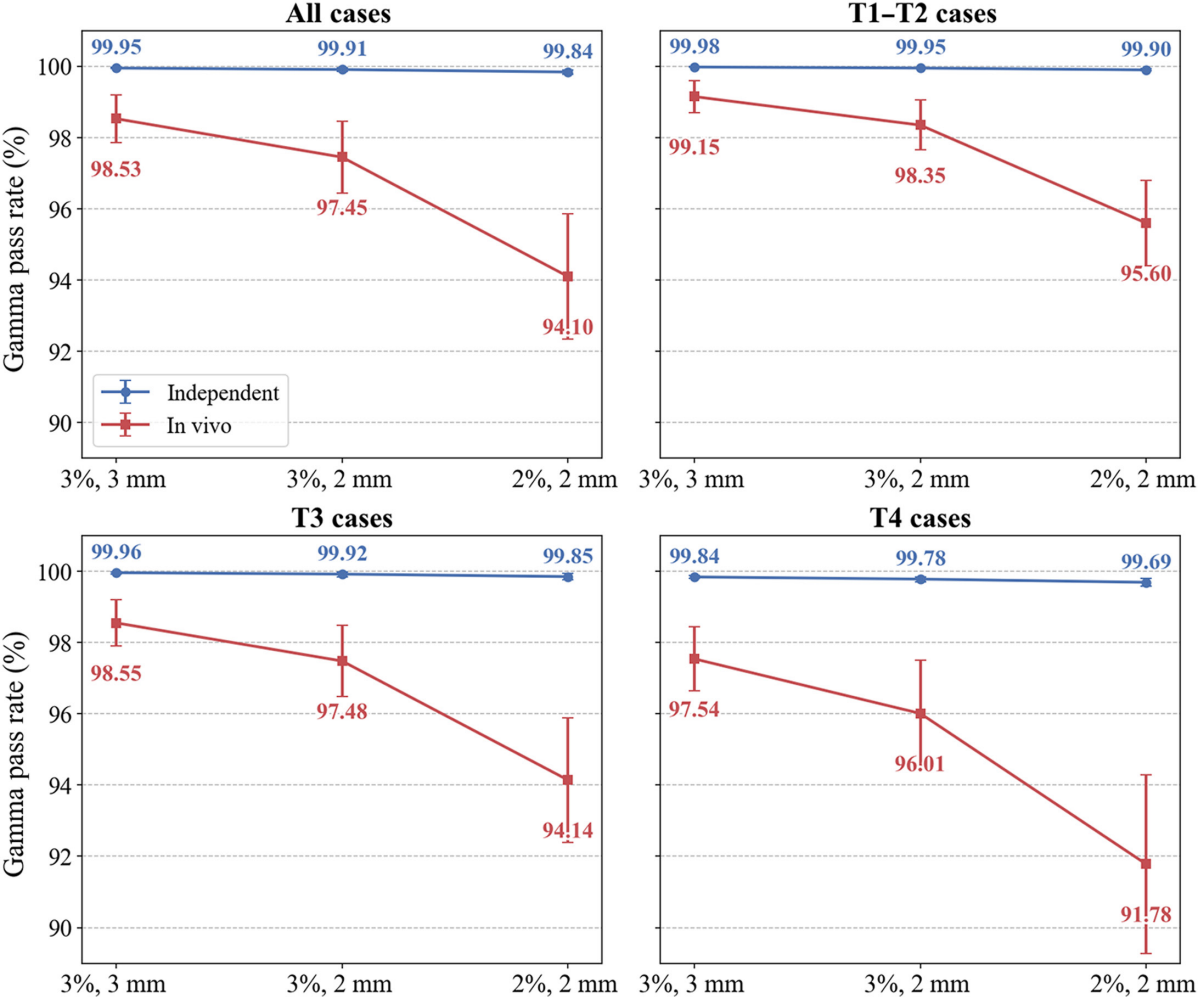
Comparison of QA outcomes between independent dose calculation and in vivo dosimetry in the prospective cohort overall and stratified by tumor stage.

Dose verification further confirmed the reliability of the planning and delivery processes across stages, and all plans met the predefined acceptance thresholds (see the “Dosimetric QA” section). As shown in Fig. 6, independent dose recalculation achieved consistently high gamma passing rates in the overall cohort and in all tumor stage subgroups. Even under the strictest 2%/2-mm criterion, the lowest pass rate was observed in the T4 subgroup and still reached 99.69%. In contrast, in vivo EPID-based transit dosimetry produced slightly lower pass rates. The overall pass rate under the 3%/3-mm criterion was 98.53%, and this modest reduction persisted after stratification, with 99.15% for T1 to T2, 98.55% for T3, and 97.54% for T4. This difference is expected because in vivo verification is more sensitive to anatomical variation, setup uncertainty, intrafraction motion, and delivery-related machine factors [38,39].

Overall, the prospective results confirm clinical readiness with high efficiency, stable dosimetry, and reliable verification in a real-time online workflow.

## Discussion

Online real-time AIO radiotherapy workflows have emerged as a promising paradigm to accelerate conventional radiotherapy by integrating simulation, contouring, planning, and delivery into a single session, thereby improving treatment efficiency and precision [13–15]. The persistent barrier is plan generation within minutes while preserving target coverage and OAR limits. While recent advances in AI-based planning have demonstrated potential in research and offline settings [29,30], few systems have achieved the necessary combination of speed, robustness, and clinical reliability for prospective deployment in real-world real-time workflows.

We addressed this need with an automated planning model trained on a large cohort, with emphasis on real-time performance, cross-institutional adaptability, and clinical feasibility. The system underwent iterative refinement through 4 major development cycles, each addressing specific challenges such as target–OAR dose trade-offs, computational latency, and anatomical complexity in T4 stage disease. Several key innovations underpinned this evolution. The use of quantile loss improved robustness to outliers in dose prediction [32,33]. Expansion of training data with additional T4 cases enhanced performance in geometrically challenging scenarios, incorporation of stochastic platform optimization to improve dose conformity and homogeneity [34], and deployment of a CT-MCDL module under a hybrid CPU–GPU architecture to support accurate, fast dose estimation [35]. Each iterative refinement was quantitatively validated, forming a transparent and interpretable trajectory of model evolution. This systematic development enhances explainability and aligns with recent recommendations on trustworthy AI in healthcare [40,41]. By directly linking algorithmic modifications to measurable clinical benefits, it fosters user trust and facilitates clinical adoption. In addition, this development pathway offers broader methodological value: As a case study of how clinical demands can guide model design, it illustrates a scalable paradigm applicable to other AI tasks such as autosegmentation and outcome modeling.

Multicenter retrospective validation across 5 institutions, including the model development center and 4 external centers, confirmed that the automated planning model generalized across different imaging protocols, contouring styles, and planning practices. The AI-generated plans not only met clinical acceptance criteria but often achieved superior target coverage and comparable OAR sparing relative to expert manual plans. Importantly, these results were obtained without institution-specific retraining or manual adjustment, underscoring the model’s potential for seamless clinical translation and interinstitutional standardization.

In a subsequent prospective deployment involving 242 patients, we conducted the largest real-world clinical validation of AI-based online planning for NPC to date. In this prospective validation, across patients of all tumor stages, the model consistently generated high-quality plans with reliable target coverage and competitive OAR sparing. It achieved a 95% acceptance rate after a single automated optimization cycle and reduced the mean optimization time to 3.5 min, compared to 11.92 min reported for prior automated planning models [42], resulting in a 70.6% improvement. This efficiency gain not only minimizes treatment delays but also reduces patient discomfort and intrafraction motion, both of which are major contributors to geometric uncertainty during online treatments and directly influence the accuracy of radiotherapy delivery [43,44]. Furthermore, all plans passed both independent secondary dose verification and EPID-based in vivo QA, reinforcing the safety and dosimetric reliability of this AI-driven workflow under routine conditions.

Our results extend prior work on automated and knowledge-based planning [20–25,29,30]. Earlier systems improved consistency yet often remained offline or site specific. Prospective evidence has been limited. Here, both retrospective benchmarking and real-world deployment showed stable performance without local retraining. This suggests portability and supports scale across institutions. Clinical impact is practical. Shorter planning time not only reduces immobilization and the chance of intrafraction motion but also enhance geometric accuracy during delivery [43,44]. Reliable first-pass plans can reduce replanning and streamline clinical physicist and physician time. Standardized priorities also limit variation in plan quality across planners and centers [16–19]. These effects are critical in regions with high patient volume but limited access to experienced planners, where scalable automation could democratize high-quality care.

Notably, while the model operates in a fully automated manner, it incorporates a priority-based customization mechanism that enables clinical experts to manually adjust target weights prior to optimization based on patient-specific anatomical considerations or clinical priorities. This flexible feature allows expert intervention early in complex scenarios, for example, when the spatial proximity between tumors and critical OARs requires nuanced dose trade-offs or when prior treatments and comorbidities call for individualized protection strategies. This hybrid design, which combines standardized automation with user-driven adaptability, provides a practical model of human–AI collaboration. It maintains planning consistency while enabling expert input for individualized optimization, aligning more closely with clinical intent and improving user acceptability, especially in complex or borderline cases where rigid automation may fall short.

Nevertheless, several limitations warrant consideration. First, while the model consistently met institutional planning criteria, doses to secondary OARs such as the cochleae, optic chiasm, and temporomandibular joints were modestly elevated in some cases. These remained within tolerance but highlight the need for further refinement to align with the ALARA (as low as reasonably achievable) principle, especially considering long-term survivorship and quality-of-life considerations [5,6,45]. Second, this study focused on initial treatment planning, and performance in adaptive replanning scenarios remains to be evaluated [46,47]. Third, although the model’s dosimetric robustness was supported by the offline multicenter benchmark, the prospective online AIO deployment was conducted at a single institution and therefore cannot be interpreted as definitive evidence of cross-center scalability of the end-to-end workflow. While dosimetric and QA end points were robust in routine clinical use, long-term oncologic outcomes, toxicity profiles, and quality-of-life metrics must be prospectively monitored to fully characterize the clinical benefits and risks of AI-based planning.

## Conclusion

We developed and clinically validated an automated planning model for NPC that works in real time within an online workflow. Iterative improvements addressed dose trade-offs, anatomic complexity, and planning speed. Multicenter benchmarking showed generalizability. A prospective deployment in 242 patients showed clinical feasibility and robustness.

The system achieved high first-pass acceptance with minimal manual input. A priority interface preserved expert oversight in complex cases. This supports scalable collaboration between clinicians and automation while maintaining individualized care.

These results support routine use in time-sensitive radiotherapy. The development-to-deployment pathway is transferable to other disease sites and supports broader adoption of standardized, fast, and safe radiotherapy.

## Data Availability

The datasets analyzed during the current study are available from the corresponding author upon reasonable request.
