# Supplementary material for: Real-Time AI-Based Radiotherapy Planning for Nasopharyngeal Carcinoma: Development and Validation
Source: Cyborg Bionic Syst. 2026 May 18;7:0544. doi: 10.34133/cbsystems.0544 (PMC13181166; doi:10.34133/cbsystems.0544)
Supplement: Supplementary 1 — Supplementary Text Tables S1 to S4 Figs. S1 to S7 [file cbsystems.0544.f1.docx]

**Supplementary Materials**

**1 Model Implementation Environment and Training Configuration**

The channel-attention densely-connected 3D (CAD-3D) UNet model was developed in PyTorch version 1.6 using Python 3.7 with CUDA 10.2 on Ubuntu 18.04. The model was trained for 1,000 epochs using the Adam optimizer with an initial learning rate of 0.0001 on an NVIDIA RTX 3090 GPU with 24 GB VRAM. The dataset included 210 training cases, 20 validation cases, and 30 test cases.

The CT-based Monte Carlo dose learning module (CT-MCDL) was implemented in TensorFlow with CUDA 10.2 on Windows. Training used the same optimizer and learning rate schedule and was run for 1,000 epochs on an NVIDIA GTX 1080 GPU with 6 GB VRAM. The dataset included 450 training cases, 30 validation cases, and 150 test cases. Testing was performed on a treatment planning system equipped with an NVIDIA GTX 1080 GPU with 8 GB VRAM.

For baseline characterization, validation and test cases were combined as a held-out set, as summarized in Table S1.

**Table S1** Baseline characteristics of model development cohorts (N = 890)

| **Characteristic** | **Dose prediction module**  **(N = 260)** | | **CT-MCDL module**  **(N = 630)** | |
| --- | --- | --- | --- | --- |
|  | **Training set**  **(N = 210)** | **Validation & Testing set**  **(N = 50)** | **Training set**  **(N = 450)** | **Validation & Testing set**  **(N = 180)** |
| **Sex** |  |  |  |  |
| Male | 132 (62.9%) | 31 (62.0%) | 285 (63.3%) | 113 (62.8%) |
| Female | 78 (37.1%) | 19 (38.0%) | 165 (36.7%) | 67 (37.2%) |
| **Age** (years) |  |  |  |  |
| Median (Range) | 48 (21~76) | 47 (23~72) | 49 (18~81) | 48 (20~79) |
| **KPS** |  |  |  |  |
| 70-80 | 0 (0) | 0 (0) | 0 (0) | 0 (0) |
| 90-100 | 210 (100%) | 50 (100%) | 450 (100%) | 180 (100%) |
| **Tumor category*** | |  |  |  |
| T1 | 57 (27.1%) | 13 (26.0%) | 44 (9.8%) | 19 (10.6%) |
| T2 | 63 (30.0%) | 17 (34.0%) | 69 (15.3%) | 28 (15.6%) |
| T3 | 41 (19.5%) | 9 (18.0%) | 204 (45.3%) | 83 (46.1%) |
| T4 | 49 (23.3%) | 11 (22.0%) | 133 (29.6%) | 50 (27.8%) |
| **Nodal category*** | |  |  |  |
| N0-1 | 131 (62.4%) | 30 (60.0%) | 286 (63.6%) | 116 (64.4%) |
| N2-3 | 79 (37.6%) | 20 (40.0%) | 164 (36.4%) | 64 (35.6%) |
| **Stage*** |  |  |  |  |
| I-II | 92 (43.8%) | 23 (46.0%) | 83 (18.4%) | 35 (19.4%) |
| III-IV | 118 (56.2%) | 27 (54.0%) | 367 (81.6%) | 145 (80.6%) |
| **Treatment modality** | |  |  |  |
| RT | 33 (15.7%) | 7 (14.0%) | 52 (11.6%) | 21 (11.7%) |
| CCRT | 83 (39.5%) | 21 (42.0%) | 155 (34.4%) | 63 (35.0%) |
| IC+CCRT | 41 (19.5%) | 11 (22.0%) | 109 (24.2%) | 43 (23.9%) |
| CCRT+AC; IC+CCRT+AC | 53 (25.2%) | 11 (22.0%) | 134 (29.8%) | 53 (29.4%) |

Abbreviations: CT-MCDL, CT-based Monte Carlo dose learning; KPS, Karnofsky performance score; RT, radiotherapy; CCRT, concurrent chemoradiotherapy; IC, induction chemotherapy; AC, adjuvant chemotherapy.

* American Joint Committee on Cancer / Union for International Cancer Control, 8th edition

**2 The role and creation method of auxiliary structure**

**2.1 Definition of formula symbol**

- ⊕: ‌Morphological dilation.
- *B*(*r*): A 3D spherical structuring element with radius *r.*
- *L*_Anterior_​(*d*): A line segment structuring element extending *d* along the Anterior direction‌.
- ***−*** : ‌Set difference‌.
- *∩* : ‌Intersection‌.
- *∪* : Union.

**2.2 Role and generation Rule of *Z40***

The creation rule of *Z40* can be formulated as:

$Z40=\left( \left( \text{PCTV2}\oplus B\left( 19 \text{mm} \right) \right)-\left( \text{PCTV2}\oplus B\left( 9 \text{mm} \right) \right) \right)\cap\left( \text{(SpinalCord}\oplus B\left( 3 \text{mm} \right){)\oplus L}_{\text{Anterior}}\left( 100 \text{mm} \right) \right)$*.*


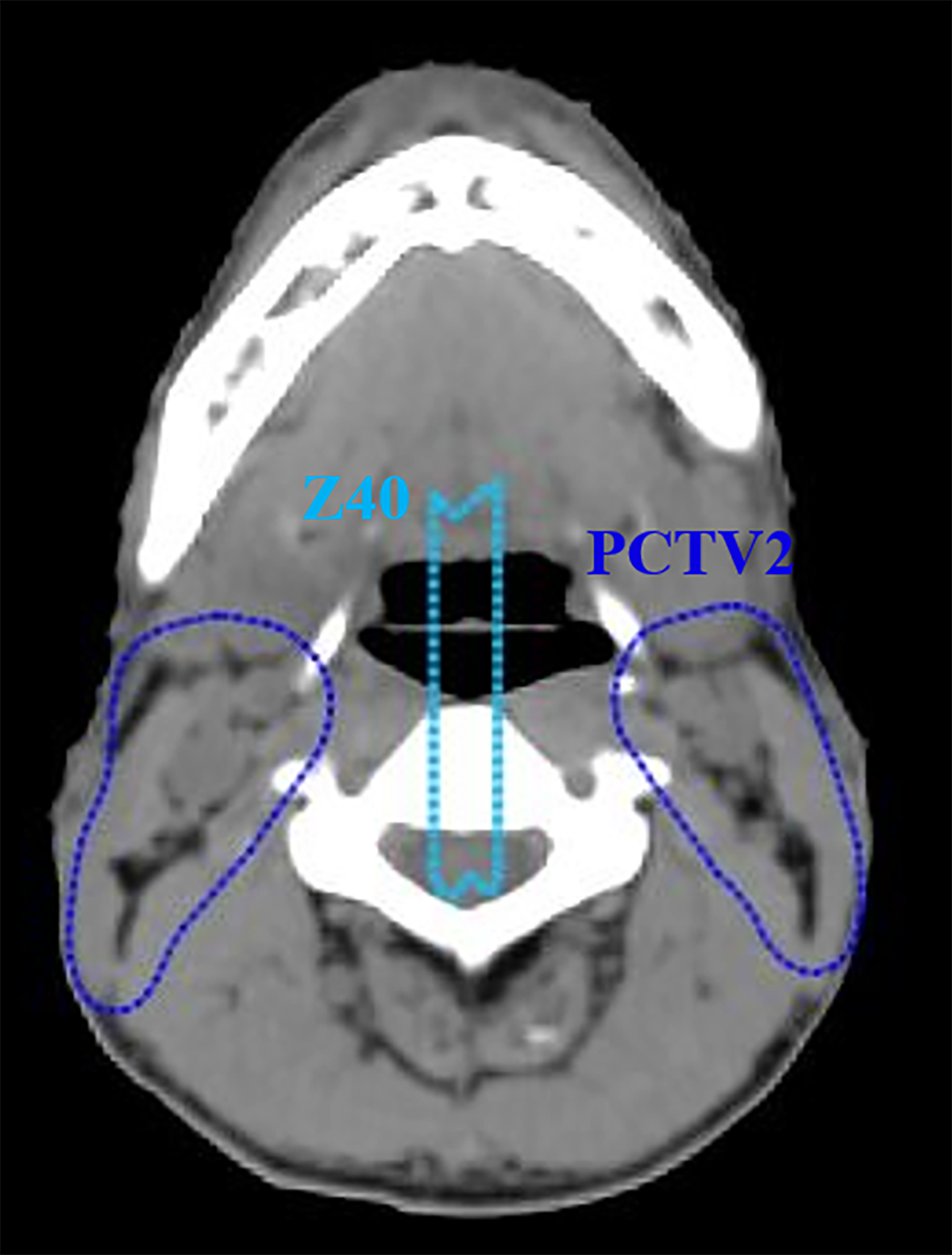


**Fig S1.** Spatial positioning of the auxiliary structure Z40 relative to PCTV2

The auxiliary structure *Z40* is designed to mitigate unnecessary radiation exposure to central oropharyngeal tissues and reduce the incidence of acute mucosal toxicity. It is a virtual structure strategically positioned along the midline within the PCTV2 region and functions as a dose modulation constraint aimed at the 4000 cGy isodose distribution. By imposing an upper dose limit of 3900 cGy on *Z40*, the optimization process is steered to deliberately disrupt the continuity of the 4000 cGy isodose line across the midline, thereby avoiding bilateral dose overlap over sensitive oropharyngeal mucosa.

**2.3 Generation Rule of *Z54_total* and *Z54_lobe***

**2.3.1 Definition of Intermediate Variables**

(1) Set ***A***‌ (generated by merging multiple expanded structures):

$A=\left( \text{PCTV2}\oplus B\left( 3 \text{mm} \right) \right)\cup\left( \text{(PGTVp}\cup\text{PGTVn\_L}\cup\text{PGTVn\_R)}\oplus B\left( 7 \text{mm} \right) \right)\cup\left( \text{(PCTV1}\cup\text{PCTVn\_L}\cup\text{PCTVn\_R)}\oplus B\left( 5 \text{mm} \right) \right)$*.*

(2) Set ***B***‌ (expanded only from PCTV2):

$B=\text{PCTV2}\oplus B\left( 10 \text{mm} \right)$*.*

**2.3.2 Definition of *Z54_total‌***

*Z54_total* is the ‌set difference between ***B*** and ***A***‌ (i.e., regions in B not overlapping with A):

$\text{Z54\_total}=B-A=\left( \text{PCTV2}\oplus B\left( 10 \text{mm} \right) \right)-A$*.*

‌
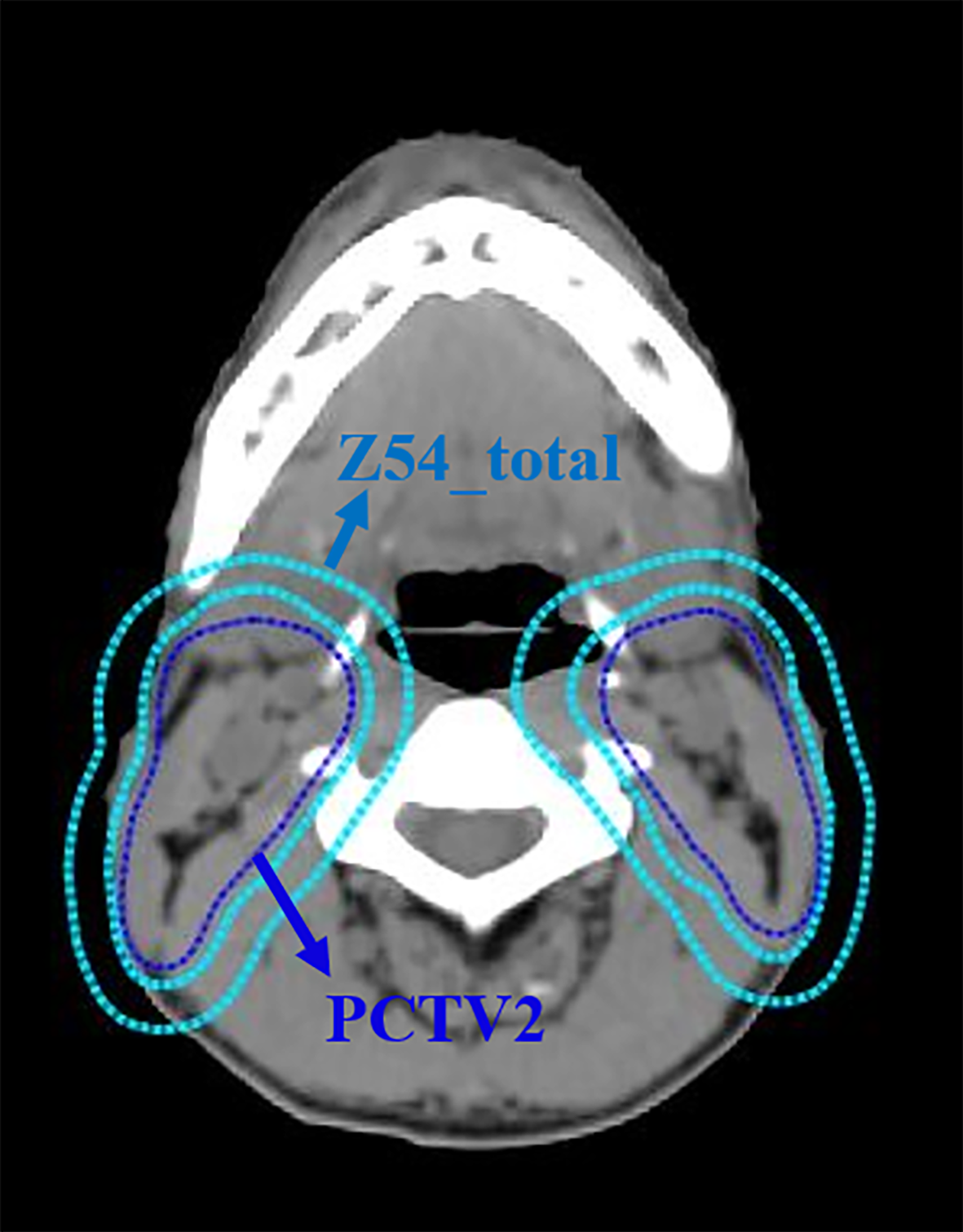


**Fig S2.** Spatial positioning of the auxiliary structure Z54_total‌ relative to PCTV2

The auxiliary structure *Z54_total* is designed to control the dose conformity of the 5400 cGy isodose line around the PCTV2 region, without compromising the high-dose coverage required for embedded target subvolumes such as PGTVp and PGTVn. Geometrically, *Z54_total* is constructed as a ring-like shell between the expanded PCTV2 volume (10 mm dilation) and the union of all high-dose target volumes with their respective margins, including PGTVp, PGTVn (bilateral), PCTV1, PCTV2, and PCTVn (bilateral). A maximum dose constraint of 5350 cGy is applied to *Z54_total*, guiding the optimizer to avoid excessive dose spillage into the peripheral regions of PCTV2. This promotes tighter dose falloff and improves the overall conformity of the intermediate dose level, particularly relevant in protecting surrounding normal tissues.

**2.3.3 Definition of *Z54_lobe*‌**

*Z54_lobe* is the ‌intersection of *Z54_total* and the Temporal Lobe‌:

$\text{Z54\_lobe}=\text{Z54\_total}\cap\text{Temporal\_Lobe}=\left( B-A \right)\cap\text{Temporal\_Lobe}$*.*


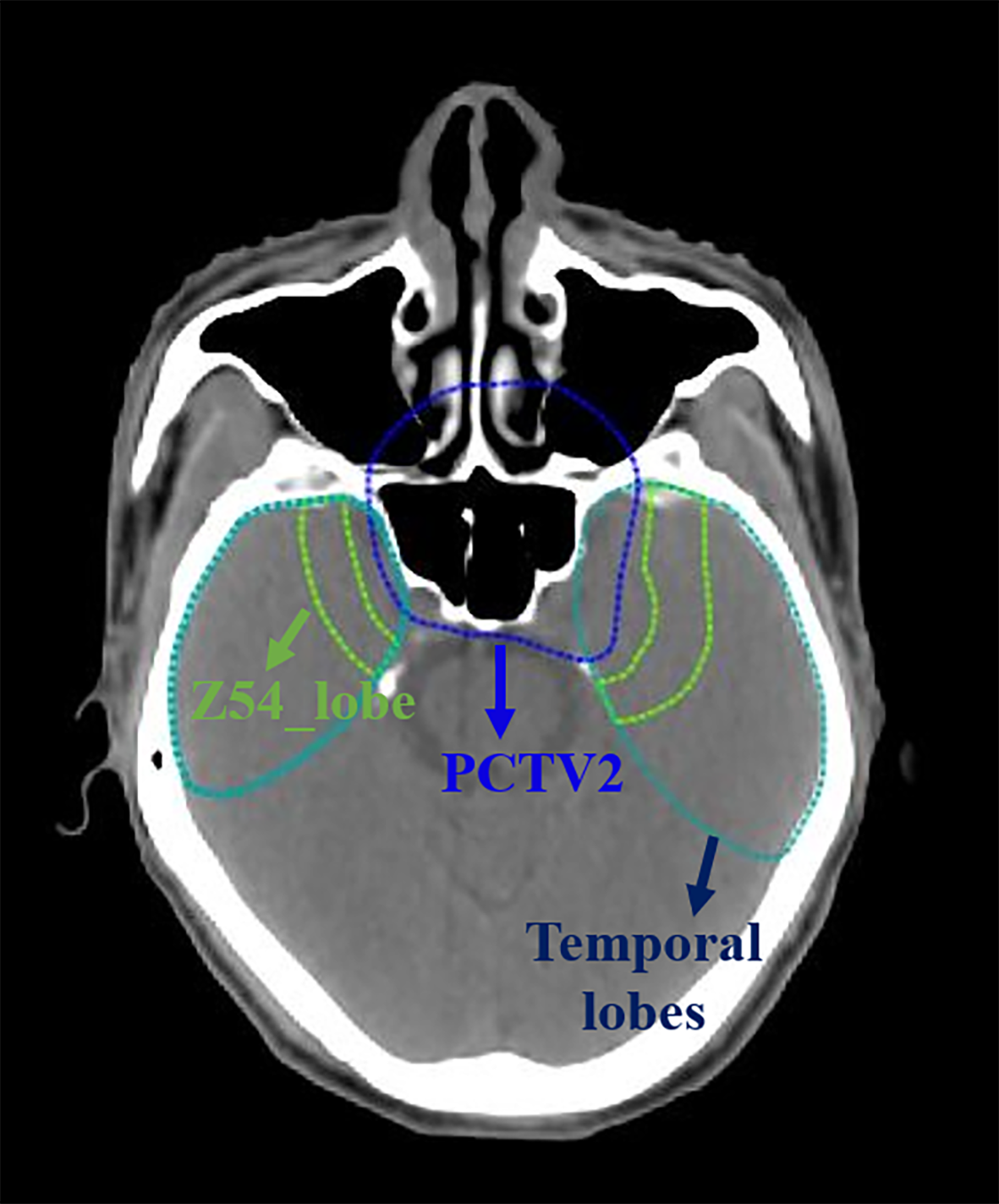


**Fig S3.** Spatial positioning of the auxiliary structure Z54_lobe ‌relative to surrounding structures

The auxiliary structure *Z54_lobe* is defined as the intersection of *Z54_total* with the bilateral temporal lobes. Its primary role is to selectively restrict the 5400 cGy dose conformity in the regions of PCTV2 that are adjacent to the temporal lobes, which are critical organs at risk (OARs) in nasopharyngeal carcinoma radiotherapy. By imposing a dose limit of D_max_ < 5400 cGy on *Z54_lobe*, the planning system is explicitly instructed to prevent unnecessary intermediate dose spill into the temporal lobes, thereby reducing the risk of radiation-induced temporal lobe injury. This structure enables spatially selective modulation of dose conformity, improving OAR sparing while maintaining robust target coverage.

**2.4 Generation Rule of *6996-BrainStem***

The creation rule of $\text{6996-Brainstem}$ can be formulated as:

$\text{6996-Brainstem}=PGTVp-\left( \text{Brainstem}\oplus B\left( 6 \text{mm} \right) \right)$*.*


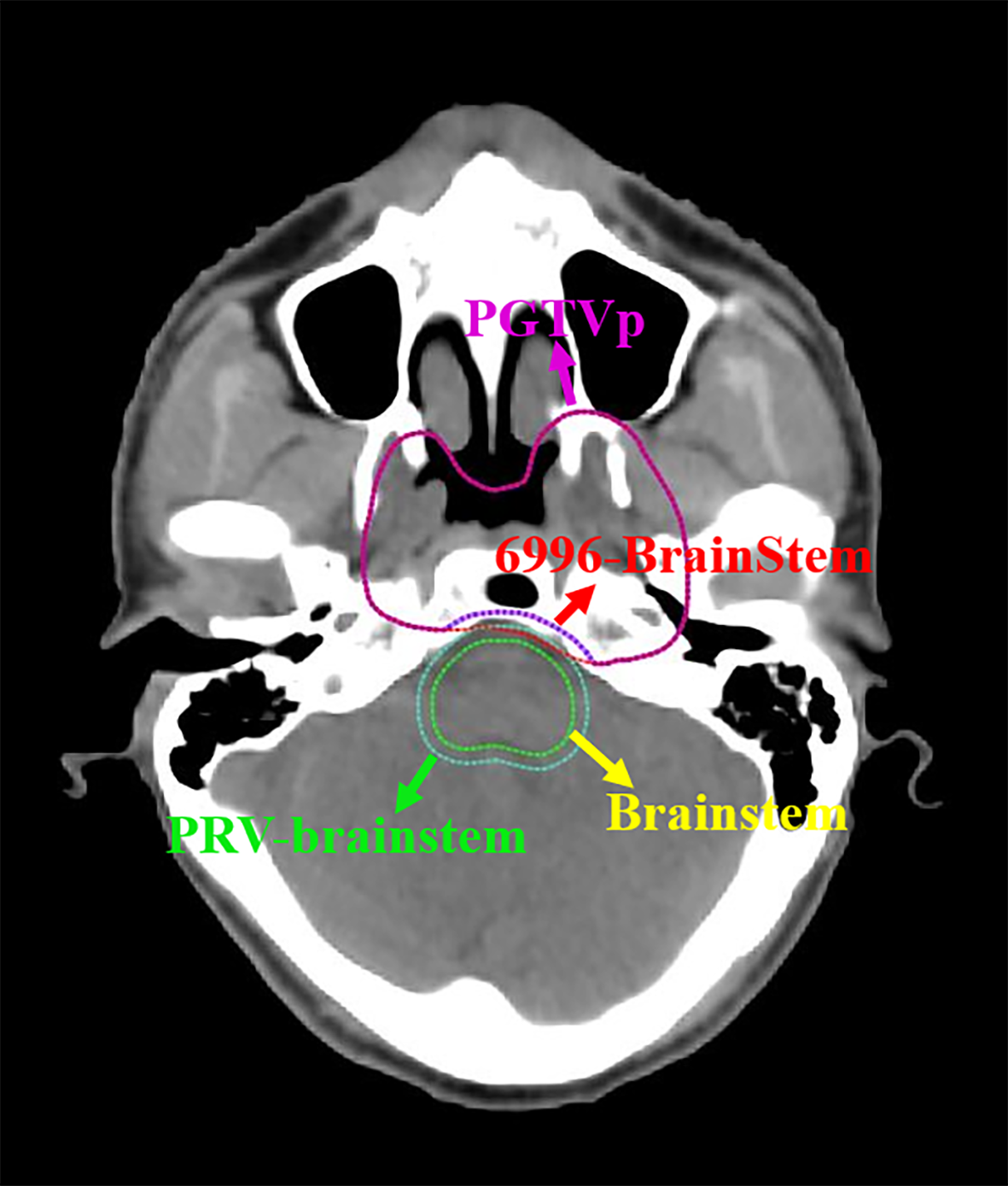


**Fig S4.** Spatial positioning of the auxiliary structure $\text{6996-Brainstem}$ ‌relative to surrounding structures

The auxiliary structure *6996-BrainStem* is defined as the subtraction of the brainstem with a 6 mm isotropic expansion from the PGTVp volume. This structure serves as a compromise zone when the high-dose target (PGTVp) is spatially adjacent to or overlapping the brainstem. By enforcing a minimum dose constraint of D_min_ ≥ 7050 cGy on this structure, the optimizer is compelled to maintain adequate dose coverage within the PGTVp subregion that is adjacent to the brainstem, while allowing for controlled compromise in less critical regions. This approach allows for adaptive sparing of the brainstem, aiming to minimize both the maximum dose and the volume receiving ≥ 6000 cGy, while preserving sufficient PGTVp coverage where feasible. *6996-BrainStem* thus provides a mechanism for intelligent trade-off between target coverage and brainstem protection in anatomically challenging cases.

**2.5 Generation Rule of *Parotid_Norm_L* and *Parotid_Norm_R***

The creation rule of *Parotid_Norm_L* and *Parotid_Norm_R* can be formulated as:

$$Parotid\_Norm\_L =Parotid\_L-\left( \text{PCTV2}\oplus B\left( 3 \text{mm} \right) \right)$$

$$Parotid\_Norm\_R =Parotid\_R-\left( \text{PCTV2}\oplus B\left( 3 \text{mm} \right) \right)$$

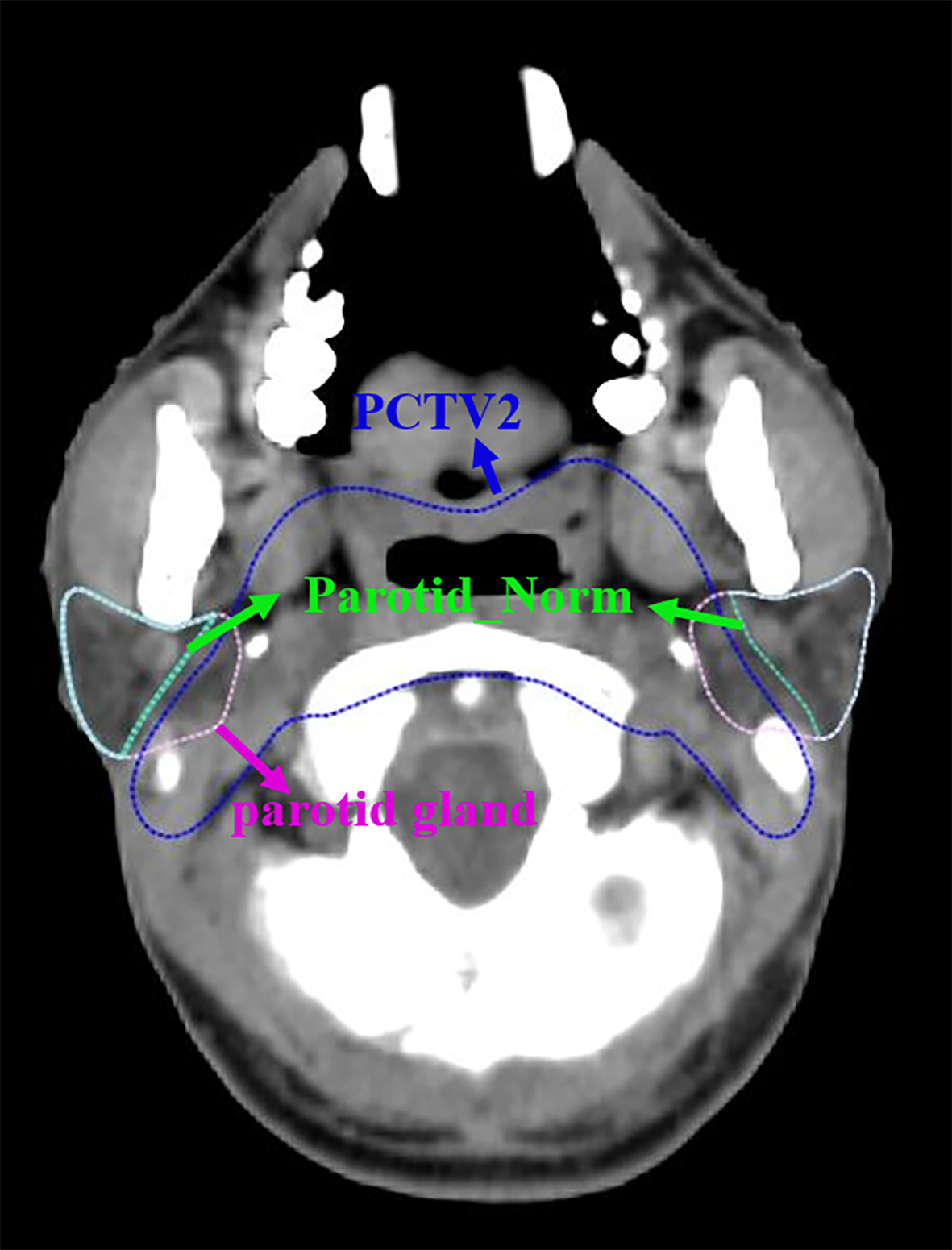


**Fig S5.** Spatial positioning of the auxiliary structure Parotid_Norm ‌relative to surrounding structures

The auxiliary structure *Parotid_Norm_L* and *Parotid_Norm_R* are generated by subtracting a 3 mm expanded PCTV2 volume from the respective ipsilateral parotid glands. These structures are designed to isolate the portion of the parotid gland outside the high-dose region, and thus represent the true dose-sparing region of the parotids. A mean dose constraint of D_mean_ ≤ 5000 cGy is applied to each, directing the optimizer to reduce radiation dose specifically in the spared region, while allowing for higher dose within regions that are in close proximity to or overlapping with the target. This selective constraint strategy enhances parotid sparing without compromising target coverage, thereby reducing the risk of xerostomia and preserving long-term salivary function.

**2.6 Generation Rule of *Ring***

**2.6.1 Definition of Intermediate Variables**

Set **C**‌ (generated by merging multiple expanded structures):

$$C=\left( \text{(PGTVp }\cup\text{PGTVn\_L }\cup\text{PGTVn\_R }\cup\text{PCTVn\_L }\cup\text{PCTVn\_R }\cup\text{PCTV1)}\oplus B\left( 3 \text{mm} \right) \right)$$

**2.6.2 Definition of *Ring***

The creation rule of $\text{Ring}$ can be formulated as:

$$Ring=PCTV2-C$$

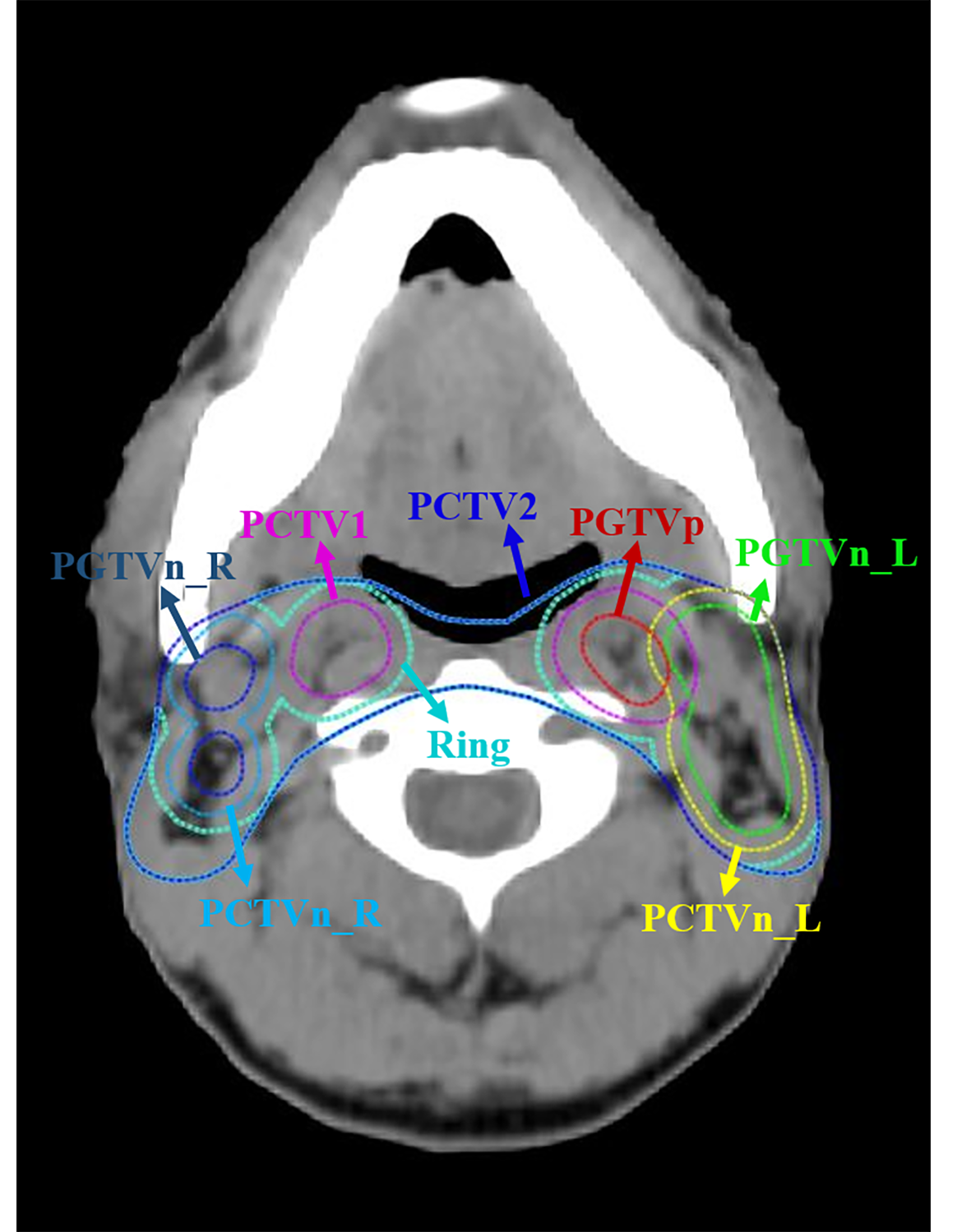


**Fig S6.** Spatial positioning of the auxiliary structure Ring ‌relative to surrounding structures

The auxiliary structure *Ring* is defined as the region within PCTV2 that lies outside a 3 mm isotropic expansion of all embedded high-dose target volumes, including PGTVp, PGTVn (bilateral), PCTVn (bilateral), and PCTV1. This structure is used to evaluate and constrain the spatial conformity of the 6000 cGy dose within the PCTV2 volume. Specifically, it targets areas of PCTV2 that are not directly adjacent to high-dose subtargets, where excessive intermediate dose deposition may indicate poor conformity or unnecessary dose spread. A dose-volume constraint of V_60Gy_ ≤ 15% is applied to the *Ring*, guiding the optimizer to suppress 6000 cGy dose spill in peripheral, low-risk regions of PCTV2. This helps to enhance the overall dose gradient sharpness and prevents the formation of high-dose "plateaus" or islands in non-critical regions, contributing to improved sparing of surrounding normal tissues without compromising high-dose target coverage.

**3 Detailed Results for Offline Benchmarks**

**3.1 Time-isolated internal benchmark:** **Developing center**

In this section, we present a comprehensive comparison between the AI-generated plans and manual plans for the developing center (n = 125). The analysis focuses on key dosimetric metrics for both targets and OARs. Quantitative measures for target coverage, including the percentage of volume receiving 100% of the prescribed dose (V_100%_) and the near-maximum dose (D_0.03cc_) for serial OARs, as well as the mean dose (D_mean_) for parallel OARs, are reported for each plan type.

Table S2 presents the detailed dosimetric results, where each row compares the AI-generated plans to manual plans across various dosimetric endpoints. The data shows consistent target coverage across both plan types, with the AI plans demonstrating slight improvements in several critical areas, particularly for targets such as PGTVp, PCTV1 and PCTV2. However, for some OARs, the results are more variable, with the AI plans performing better in some instances (e.g., parotid glands), while manual plans excelled for others (e.g., cochleae).

These results demonstrate that the AI-generated plans provide comparable or superior coverage of clinical targets, along with competitive OAR sparing, underscoring the potential for automated planning in routine clinical practice.

**Table S2** Quantitative dosimetric comparison between AI and manual plans in the time-isolated internal test cohort from the developing center (n = 125)

| **ROIs** | **Metrics** | **Criteria**  **(cGy)** | **Developing center** | |
| --- | --- | --- | --- | --- |
|  |  |  | **AI plans** | **Manual plans** |
| **Targets** |  |  |  |  |
| PGTVp | V_100%_ † | ≥ 95% | 98.9±0.9* | 98.5±1.3 |
|  | V_98%_ † | / | 99.6±0.5 | 99.6±0.5 |
|  | D_100%_ § | / | 6527.24±253.58 | 6475.35±408.63 |
|  | D_98%_ ‡ | / | 7056.13±73.03* | 7028.21±73.44 |
| PGTVn | V_100%_ | ≥ 95% | 99±0.8 | 99±1.0 |
|  | V_98%_ | / | 100±0.1 | 100±0.1 |
|  | V_95%_ ♯ | / | 99.9±0 | 99.9±0 |
| PCTV1 | V_100%_ | ≥ 95% | 99.9±0.3* | 99.7±0.4 |
|  | V_98%_ | / | 100±0 | 100±0 |
|  | D_100%_ | / | 5754.3±63.24* | 5591.12±190.84 |
|  | D_98%_ | / | 6282.87±63.7* | 6208.68±40 |
| PCTV2 | V_100%_ | ≥ 95% | 98.7±0.8* | 98.4±1.0 |
|  | V_98%_ | / | 100±0 | 100±0.1 |
|  | D_100%_ | / | 4659.42±158.48 | 4579.2±222.19 |
|  | D_98%_ | / | 5469.8±69.06* | 5433.2±48.76 |
| **Organs at risk** |  |  |  |  |
| Spinal cord | D_0.03cc_ & | ≤ 5000 | 3577.07±356.09* | 3465.17±356.67 |
| Brain stem | D_0.03cc_ | ≤ 6000 | 5742.92±708.24 | 5740.37±700.39 |
| Temporal lobes | D_0.03cc_ | ≤ 7200 | 7139.93±419.62* | 7186.28±419.88 |
| Optic chiasm | D_0.03cc_ | ≤ 6000 | 4066.39±1922.46 | 4101.09±2093.43 |
| Optic nerves | D_0.03cc_ | ≤ 6000 | 4120.37±1893.62* | 4202.56±1994.6 |
| Eyes | D_mean_ ^ | < 3500 | 834.16±344.25* | 757.75±279.19 |
| Lenses | D_0.03cc_ | ≤ 600 | 664.63±211.88* | 583±146.59 |
| Cochleas | D_mean_ | < 5500 | 5113.12±728.51* | 4904.58±849.08 |
| Parotid glands | D_mean_ | < 3000 | 3418.09±427.59* | 3626.76±360.69 |
| Submandibular glands | D_mean_ | < 3500 | 5317.34±398.29 | 5301.95±502.7 |
| Pituitary | D_max_ @ | < 6000 | 6002.02±1367.34 | 5913.77±1391.46 |
| Thyroid | D_mean_ | < 4500 | 4476.56±671.3 | 4477.66±674.12 |
| Oral cavity | D_mean_ | < 4000 | 3414.56±286.86* | 3468.62±282.94 |
| Larynx supraglottic | D_mean_ | < 4500 | 3560.91±410.24 | 3573.31±407.84 |
| Mandible | D_0.03cc_ | < 6500 | 6953.44±508.23 | 6975.86±501.58 |
| Temporomandibular joints | D_2%_ $ | < 7500 | 5744.27±761.43 | 5685.7±874.41 |

**Note:** Prescription: PGTVp, 6996cGy/33Fr; PGTVn_L/R, 6600 or 6996 cGy/33Fr; PCTV1, 6006 cGy/33Fr; PCTV2, 5412 cGy/33Fr.

**Abbreviations:** SD, standard deviation; PGTVp, planning target volume of gross tumor at the primary site and the involved retropharyngeal lymph nodes; PGTVn, planning target volume of involved cervical lymph nodes; PCTV1, planning target volume of high-risk clinical target volume; PCTV2, planning target volume of low-risk clinical target volume; ROIs, regions of interest.

*: *p* ＜ 0.05

†: Percent of volume receiving ≥ 100% of the prescribed dose.

†: Percent of volume receiving ≥ 98% of the prescribed dose.

§: Dose received by 100% of the target volume.

‡: Dose received by 98% of the target volume.

♯: Percent of volume receiving ≥ 95% of the prescribed dose.

&: Dose received by 0.03cm^3^ of the volume.

^: Mean dose to the volume.

@: Maximum dose to the volume.

$: Dose received by 2% of the volume.

**3.2 External multicenter benchmark: Four independent centers**

In this section, we extend our analysis to four independent external centers, where we perform a similar comparison between AI-generated plans and manual plans (n = 120, 30 patients per center). This multicenter validation cohort enables us to evaluate the generalizability of the AI model across different clinical settings. Table S3 presents the full quantitative dosimetric results for each of the external centers.

The dosimetric comparison covers both targets and OARs, with results showing that AI plans generally maintain high target coverage and comparable OAR sparing across all external centers. However, variations are noted between centers, especially with regard to specific OARs. For example, in some centers, the AI plans demonstrated statistically significant improvements in sparing certain critical OARs (e.g., spinal cord, thyroid), while in other centers, manual plans showed superior performance, especially for organs like the cochleas.

Figure 5 in the main text summarizes the relative differences between AI and manual plans, highlighting where AI plans performed significantly better or worse. This figure allows for a quick visual comparison of AI vs. manual performance across the four centers, complementing the numerical data provided in Table S3.

In conclusion, the results from the external validation centers further support the robustness and adaptability of the AI model across a variety of clinical environments, reaffirming its potential for widespread clinical use in NPC radiotherapy.

**Table S3** Quantitative dosimetric comparison between AI and manual plans across four independent external centers (n = 120, 30 per center)

| **ROIs** | **Metrics** | **Criteria**  **(cGy)** | **External center Ⅰ** | | **External center Ⅱ** | | **External center Ⅲ** | | **External** **center** **Ⅳ** | |
| --- | --- | --- | --- | --- | --- | --- | --- | --- | --- | --- |
|  |  |  | **AI plans** | **Manual plans** | **AI plans** | **Manual plans** | **AI plans** | **Manual plans** | **AI plans** | **Manual plans** |
| **Targets** |  |  |  |  |  |  |  |  |  |  |
| PGTVp | V_100%_ † | ≥ 95% | 98.9 ± 1.1 | 98.5 ± 1.2 | 98.9 ± 1.2* | 95.5 ± 1 | 98.4±0.8* | 97.7±0.6 | 99.2±0.7* | 99.5±0.9 |
|  | V_98%_ † | / | 99.9 ± 0.2 | 99.9 ± 0.2 | 99.8 ± 0.5* | 99.3 ± 0.6 | 99.9 ± 0.1 | 99.7 ± 0.3 | 99.8±0.2 | 99.6±0.7 |
|  | D_100%_ § | / | 6718.3 ± 208.3 | 6656.8 ± 228.2 | 6712.6 ± 513.8* | 6451.8 ± 356.3 | 6690.45±146.58 | 6619.55±258.68 | 6533±228.04 | 6485.39±813.59 |
|  | D_98%_ ‡ | / | 6918.3 ± 149 | 6875.9 ± 185.8 | 7138.2 ± 325.5* | 6935.6 ± 36.8 | 7013.94±31.55* | 6986.16±19.52 | 7006.29±25.97 | 7029.38±103.72 |
| PGTVn | V_100%_ | ≥ 95% | 99.3 ± 0.9 | 99.5 ± 0.9 | 98.7 ± 1.2* | 95.5 ± 1.3 | 98.7±1 | 98.4±1.6 | 98.6±0.4* | 99.9±0.1 |
|  | V_98%_ | / | 100 ± 0 | 100 ± 0 | 99.8 ± 0.6* | 99.4 ± 0.4 | 99.9±0.2 | 99.8±0.3 | 99.7±0.2* | 100±0 |
|  | V_95%_ ♯ | / | 100 ± 0 | 100 ± 0 | 99.9 ± 0.5 | 100 ± 0.1 | 100±0.1 | 100±0 | 100±0.1* | 100±0 |
| PCTV1 | V_100%_ | ≥ 95% | 99.1 ± 0.8 | 99.1 ± 1 | 99.4 ± 0.9 | 99.1 ± 0.5 | 99.5±0.8 | 98.2±2.4 | 99.8±0.3* | 99.9±0.1 |
|  | V_98%_ | / | 99.8 ± 0.3 | 99.8 ± 0.2 | 99.6 ± 0.8 | 99.7 ± 0.4 | 99.8±0.5 | 99.2±1.6 | 99.9±0.1 | 100±0 |
|  | D_100%_ | / | 5760.6 ± 149.8 | 5706.6 ± 233.3 | 5637.2 ± 783.7 | 5418.3 ± 755.6 | 5399.87±847.22 | 5399.38±468.83 | 5437.59±1167.82 | 5437.11±818.22 |
|  | D_98%_ | / | 6056.2 ± 43.9 | 6082.2 ± 76 | 6339.9 ± 287* | 6172 ± 39.6 | 6172.46±93.89* | 6054.72±122.05 | 6156.02±41.46* | 6216.37±43.56 |
| PCTV2 | V_100%_ | ≥ 95% | 98.4 ± 0.7 | 98.2 ± 1 | 99.9 ± 0.3* | 97 ± 0.9 | 98.1±0.5 | 98.3±1.1 | 98.9±0.5* | 99.4±0.4 |
|  | V_98%_ | / | 99.6 ± 0.3 | 99.4 ± 0.5 | 99.9 ± 0.3* | 99 ± 0.4 | 99.3±0.3 | 99.4±0.7 | 99.6±0.3 | 99.8±0.2 |
|  | D_100%_ | / | 4597.6 ± 246.1 | 4416.1 ± 516.5 | 5026.6 ± 805* | 4293.1 ± 594.7 | 4346.3±836.21 | 4470.33±387.66 | 4460.49±1000.17 | 4557.67±625.37 |
|  | D_98%_ | / | 5418.8 ± 98.1 | 5386.6 ± 142.8 | 6410 ± 250.8* | 5410.5 ± 35.6 | 5414.68±23.22 | 5453.15±86.02 | 5461.18±30.72* | 5496.43±37.69 |
| **Organs at risk** |  |  |  |  |  |  |  |  |  |  |
| Spinal cord | D_0.03cc_ & | ≤ 5000 | 3344 ± 391.8* | 3634.6 ± 187.3 | 3446 ± 280 | 3371.9 ± 173.3 | 3502.46±228.4* | 3682.5±229.55 | 3877.73±312.59 | 3838.1±55.35 |
| Brain stem | D_0.03cc_ | ≤ 6000 | 4894.6 ± 682.1 | 4841.1 ± 436.3 | 5053.6 ± 985.4 | 4885.5 ± 712.9 | 5407.47±1158.8 | 5050.42±1078.21 | 5482.86±653.69 | 5659±304.86 |
| Temporal lobes | D_0.03cc_ | ≤ 7200 | 6228.3 ± 614.6 | 6418.5 ± 699.6 | 6617.5 ± 672.7 | 6577.1 ± 705.5 | 5620.02±2617.56 | 5539.93±2603.7 | 6833.21±746.8 | 6882.38±522.91 |
| Optic chiasm | D_0.03cc_ | ≤ 6000 | 2375.2 ± 2160.1 | 2537 ± 2128.9 | 1365.1 ± 1158.5 | 1580.7 ± 1374.8 | 2464.24±2460.41 | 2087.19±2277.15 | 2676.2±2100.88 | 2955.83±2176.19 |
| Optic nerves | D_0.03cc_ | ≤ 6000 | 2439.3 ± 2121.6 | 2604.2 ± 2079.9 | 2438.1 ± 1733.8 | 2381.1 ± 1542.9 | 2963.03±2267.69 | 2704.93±2135.73 | 2598.97±1834.31 | 2843.87±1989.77 |
| Eyes | D_mean_ ^ | < 3500 | 459.8 ± 166.7 | 513.1 ± 179.7 | 479.4 ± 170.6 | 523.2 ± 223.4 | 595.51±349.32 | 520.65±282 | 337.54±135.56 | 417.19±187.97 |
| Lenses | D_0.03cc_ | ≤ 600 | 419.3 ± 137.3 | 444 ± 111 | 410.8 ± 118.4 | 376.7 ± 117.5 | 520.6±268.7 | 422.73±190.73 | 288.13±99.09* | 369.15±152.34 |
| Cochleas | D_mean_ | < 5500 | 4182.4 ± 751.5 | 4148.3 ± 798 | 4674.1 ± 710.5* | 3825.4 ± 716.7 | 4346.74±2123.89 | 4270.92±2139.5 | 5226.88±916.41 | 5444.06±902.27 |
| Parotid glands | D_mean_ | < 3000 | 3005.9 ± 386.5* | 3297.9 ± 334.7 | 3262.7 ± 452.6* | 2960.8 ± 330.3 | 4112.29±489.25 | 3986.84±596.83 | 3373.61±332.22 | 3495.57±78.32 |
| Submandibular glands | D_mean_ | < 3500 | 4818.5 ± 383.9* | 5113.2 ± 361.3 | 4944.6 ± 650.5* | 4599.8 ± 489.3 | 5603.14±481.97 | 5258.59±660.16 | 5770.89±417.9* | 6167.99±348.77 |
| Pituitary | D_max_ @ | < 6000 | 3731.6 ± 2128.3 | 3922.5 ± 1908.6 | 3699 ± 2173 | 3971.7 ± 2039.1 | 3814.57±2729.21 | 3379.67±2797.17 | 5100.88±1995.95 | 5177.84±1896.91 |
| Thyroid | D_mean_ | < 4500 | 3627.1 ± 873.9 | 3730.5 ± 862 | 4674.8 ± 598.9* | 4053.3 ± 269.6 | 4932.85±465.06 | 4997.95±456.51 | 4720.82±229.65* | 5014.32±162.85 |
| Mandible | D_0.03cc_ | < 6500 | 6135.1 ± 501.3 | 6245.1 ± 590.9 | 6743.7 ± 792.4 | 6439.5 ± 750.3 | 6782.19±453.54 | 6811.94±506.4 | 7199.99±191.29* | 7409.47±144.46 |
| Temporomandi-bular joints | D_2%_ $ | < 7500 | 5174.3 ± 867.3 | 5270.2 ± 787.2 | 5432.1 ± 850.4 | 4990 ± 969.2 | 4983.16±2242.27 | 4975.55±2241.33 | 6209.41±536.92 | 6288.52±475.61 |

**Note:** Prescription: PGTVp, 6996cGy/33Fr; PGTVn_L/R, 6600 or 6996 cGy/33Fr; PCTV1, 6006 cGy/33Fr for external center Ⅰ, Ⅲ, and Ⅳ, 6105 cGy/33Fr for external center Ⅱ; PCTV2, 5412 cGy/33Fr for external center Ⅲ and Ⅳ, 5445 cGy/33Fr for external center Ⅰ and Ⅱ.

**Abbreviations:** SD, standard deviation; PGTVp, planning target volume of gross tumor at the primary site and the involved retropharyngeal lymph nodes; PGTVn, planning target volume of involved cervical lymph nodes; PCTV1, planning target volume of high-risk clinical target volume; PCTV2, planning target volume of low-risk clinical target volume; ROIs, regions of interest.

*: *p* ＜ 0.05

†: Percent of volume receiving ≥ 100% of the prescribed dose.

†: Percent of volume receiving ≥ 98% of the prescribed dose.

§: Dose received by 100% of the target volume.

‡: Dose received by 98% of the target volume.

♯: Percent of volume receiving ≥ 95% of the prescribed dose.

&: Dose received by 0.03cm^3^ of the volume.

^: Mean dose to the volume.

@: Maximum dose to the volume.

$: Dose received by 2% of the volume.

**4 Inter-planner variability analysis**

To quantify the intrinsic variability of clinically acceptable manual planning and to contextualize the AI–manual differences within this variability band, we performed a representative case analysis. A single NPC patient with stage T3N1M0 was selected. The planning CT and the physician-approved target volumes and OAR structure set were fixed, and the prescription and planning objectives followed the same institutional protocol as used in the study. Three independent manual plans were then generated by different planners, with each planner completing the full planning process for the same case. In parallel, an AI plan was generated for the same case using the final automated planning workflow described in the main Methods. All plans were evaluated using the same dose calculation settings and the same dosimetric endpoints as those used in the main analyses.

As shown in Table S4 and the representative dose distributions (Figure S7), target coverage was consistently high across all plans, and the AI plan fell within the manual variability range. For example, PGTVp V_100%_ ranged from 99.40% to 99.77% across the manual plans, and the AI plan achieved 99.78%. PCTV2 V_100%_ ranged from 99.36% to 99.93% across the manual plans, and the AI plan achieved 99.87%. Similar consistency was observed for other target metrics, indicating that the AI plan performance aligned with the range of expert manual solutions for clinically relevant coverage endpoints.

For OAR sparing, the manual plans exhibited substantial variability, reflecting different but acceptable trade-offs, and the AI plan remained within this manual variability range for key endpoints. For instance, optic chiasm D_0.03cc_ varied from 5522.18 to 6598.21 cGy across manual plans, while the AI plan achieved 5590.71 cGy. Optic nerve D_0.03cc_ ranged from 5692.08 to 6180.39 cGy in manual plans, and the AI plan achieved 5708.12 cGy. Spinal cord D_0.03cc_ ranged from 3024.39 to 3857.05 cGy in manual plans, and the AI plan achieved 3217.23 cGy. Overall, this analysis supports that NPC planning admits multiple clinically acceptable manual solutions, and it shows that the AI–manual differences remain within the intrinsic variability of expert manual planning.

**Ta****ble S4** Dosimetric variability among three independent manual plans and an AI-generated plan for a representative T3N1M0 NPC case

| ROIs | Metrics | Criteria  (cGy) | Dosimetric Results (cGy) | | | |
| --- | --- | --- | --- | --- | --- | --- |
|  |  |  | Planer 1 | Planer 2 | Planer 3 | AI plan |
| Targets |  |  |  |  |  |  |
| PGTVp | ^*^V_100%_ | ≥ 95% | 99.77 | 99.67 | 99.40 | 99.78 |
|  | ^§^D_100%_ | / | 6703.26 | 6520.59 | 6756.21 | 6763.71 |
|  | ^‡^D_98%_ | / | 7074.57 | 7111.33 | 7055.70 | 7092.77 |
| PGTVn | V_100%_ | ≥ 95% | 100 | 99.98 | 99.77 | 99.67 |
|  | ^†^V_98%_ | / | 100 | 100 | 100 | 100 |
|  | ^♯^V_95%_ | / | 100 | 100 | 100 | 100 |
| PCTV1 | V_100%_ | ≥ 95% | 100 | 100 | 99.87 | 99.98 |
|  | D_100%_ | / | 6019.18 | 5985.43 | 5833.63 | 5889.69 |
|  | D_98%_ | / | 6372.36 | 6325.35 | 6207.07 | 6250.36 |
| PCTV2 | V_100%_ | ≥ 95% | 99.68 | 99.93 | 99.36 | 99.87 |
|  | D_100%_ | / | 5074.04 | 5113.69 | 5020.82 | 5087.06 |
|  | D_98%_ | / | 5491.23 | 5547.50 | 5470.53 | 5521.01 |
| Organs at risk |  |  |  |  |  |  |
| Spinal cord | ^&^D_0.03cc_ | ≤ 5000 | 3857.05 | 3024.39 | 3182.44 | 3217.23 |
| Brain stem | D_0.03cc_ | ≤ 6000 | 6258.07 | 6134.97 | 6365.12 | 6369.27 |
| Temporal lobes | D_0.03cc_ | ≤ 7200 | 7422.31 | 7267.95 | 7400.54 | 7461.82 |
| Optic chiasm | D_0.03cc_ | ≤ 6000 | 6598.21 | 5814.26 | 5522.18 | 5590.71 |
| Optic nerves | D_0.03cc_ | ≤ 6000 | 6180.39 | 6001.55 | 5692.08 | 5708.12 |
| Eyes | ^^^ D_mean_ | ＜3500 | 985.48 | 817.36 | 810.84 | 811.42 |
| Lenses | D_0.03cc_ | ≤ 600 | 663.78 | 752.97 | 693.43 | 690.29 |
| Cochleas | D_mean_ | ＜5500 | 5586.40 | 5781.51 | 5514.55 | 5594.50 |
| Parotid glands | D_mean_ | ＜3000 | 3471.15 | 3566.15 | 3335.80 | 3464.53 |
| Submandibular glands | D_mean_ | ＜3500 | 5282.33 | 5188.20 | 4775.83 | 4847.86 |
| Pituitary | ^@^D_max_ | ＜6000 | 6223.01 | 6176.39 | 5887.68 | 6165.22 |
| Thyroid | D_mean_ | ＜4500 | 4946.70 | 4925.41 | 4348.71 | 4408.22 |
| Mandible | D_0.03cc_ | ＜6500 | 6897.95 | 7158.78 | 6785.84 | 6825.03 |
| Temporomandibular joints | ^$^D_2%_ | ＜7500 | 5579.38 | 5635.06 | 5448.11 | 5551.57 |

**Note:** Prescription: PGTVp, 6996cGy/33Fr; PGTVn_L, 6600 cGy/33Fr; PGTVn_R, 6996 cGy/33Fr; PCTV1, 6006 cGy/33Fr; PCTV2, 5412 cGy/33Fr. All data are presented as mean ± standard deviation.

**Abbreviations:** PCTV1, planning target volume of high-risk clinical target volume; PCTV2, planning target volume of low-risk-clinical target volume; PGTVn, planning target volume of involved cervical lymph node; PGTVp, planning target volume of gross tumor at the primary site and the involved retropharyngeal lymph node; ROIs, regions of interest.

*: Percent of volume receiving ≥100% of the prescribed dose.

†: Percent of volume receiving ≥98% of the prescribed dose.

§: Dose received by100% of the target volume.

‡: Dose received by 98% of the target volume.

♯: Percent of volume receiving ≥95% of the prescribed dose.

&: Dose received by 0.03cm^3 of the volume.

^: Mean dose to the volume.

@: Maximum dose to the volume.

$: Dose received by 2% of the volume.


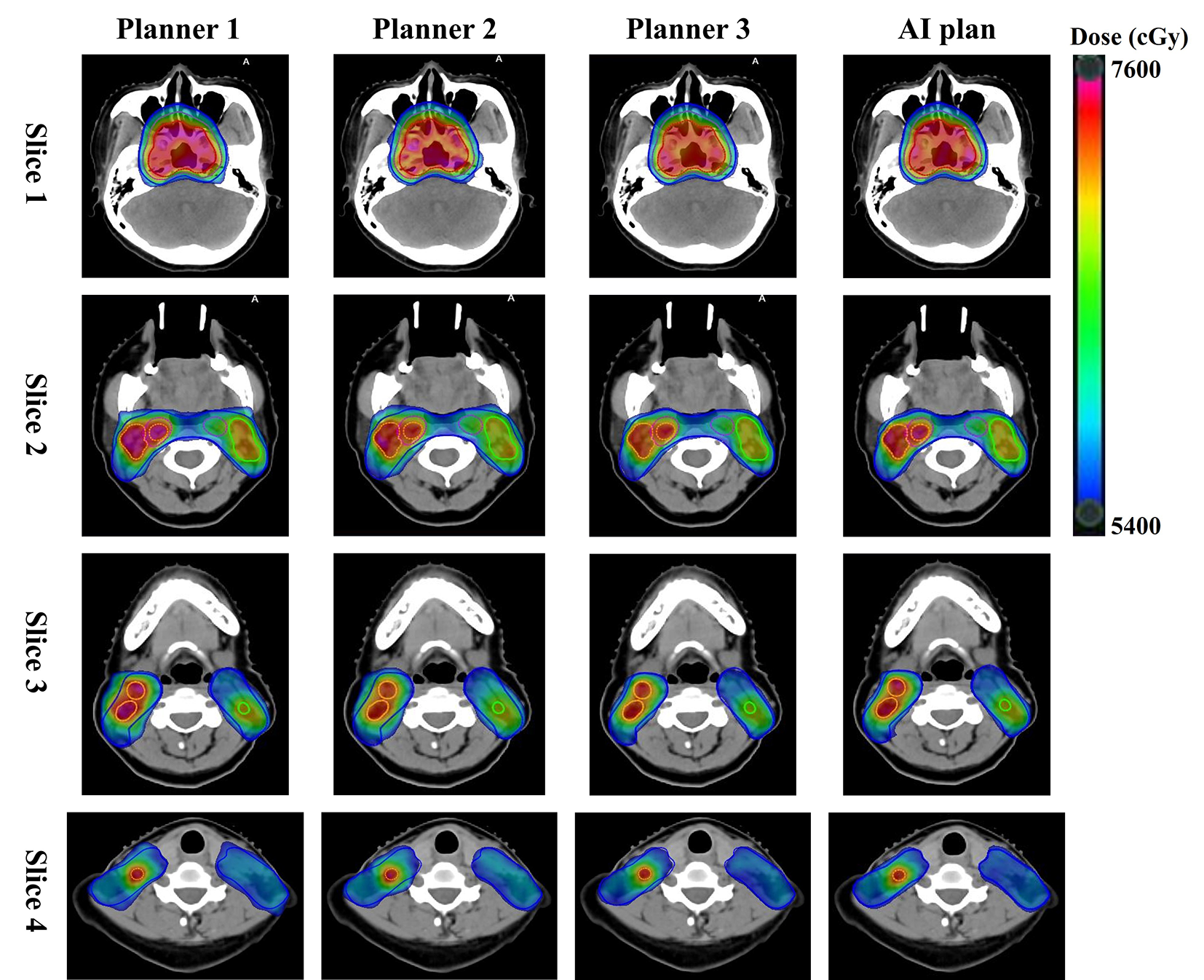


**Figure S7** Dose distributions comparing three manual plans and the AI plan for a representative T3N1M0 nasopharyngeal carcinoma case
